# Berry Consumption and Its Role in the Modulation of Obesity and Mild Cognitive Impairment

**DOI:** 10.3390/nu18040674

**Published:** 2026-02-19

**Authors:** Gustavo Alves Andrade dos Santos, Caroline Pereira Mourão Moraes, Mário Roberto Maróstica Júnior

**Affiliations:** Department of Food Science and Nutrition (DECAN), School of Food Engineering (FEA), University of Campinas (UNICAMP), Campinas 13083-862, São Paulo, Brazil; gasantos@unicamp.br (G.A.A.d.S.); c290265@dac.unicamp.br (C.P.M.M.)

**Keywords:** berries, obesity, mild cognitive impairment, anthocyanins, polyphenols

## Abstract

Most dementias are preceded by mild cognitive impairment (MCI), a transitional clinical stage characterized by cognitive decline that does not yet significantly interfere with activities of daily living. Obesity and diabetes are among the major risk factors for MCI and are strongly associated with unhealthy lifestyle patterns. The growing global prevalence of obesity has intensified the need for effective dietary strategies that promote both weight control and neuroprotection. Red fruits, which are rich in bioactive compounds such as anthocyanins, have demonstrated potential roles in modulating metabolic pathways and cognitive function. This systematic review aimed to identify and synthesize evidence from human studies published over the past two decades that examined the effects of red fruit consumption on obesity-related mechanisms and cognitive outcomes, as well as its influence on key neurodegenerative biomarkers, including TAU protein, β-amyloid, and neurofilament light chain. A systematic search was conducted in major scientific databases to identify human clinical trials evaluating the metabolic and neuroprotective effects of berry-derived compounds. Eligible studies were screened for outcomes related to cognitive performance, obesity-related parameters, and relevant molecular biomarkers. The included studies reported modest improvements in cognitive domains, with the most consistent effects observed in memory-related outcomes. Berry-derived bioactive compounds demonstrated potential in attenuating TAU protein hyperphosphorylation and reducing β-amyloid accumulation; however, the available evidence remains limited and requires further confirmation. Human clinical studies remain scarce, and although some trials reported favorable metabolic effects, these findings are still inconclusive. Reported outcomes included improvements in insulin sensitivity, regulation of leptin levels, and modulation of the gut–brain axis, which may collectively contribute to a reduced risk of obesity. Based on the studies evaluated in this review, there remains a limited number of human clinical trials that robustly support the neuroprotective and complementary metabolic effects of berry-derived bioactive compounds. Nevertheless, the available evidence suggests that dietary strategies incorporating wild fruits rich in polyphenols may represent a promising complementary approach for the prevention of mild cognitive impairment (MCI) and obesity, with potential implications for reducing the risk of dementia progression.

## 1. Introduction

Over the past few decades, the concept of mild cognitive impairment (MCI) has been refined to describe a transitional stage between normal age-related cognitive decline and dementia. Individuals with MCI exhibit cognitive deficits that exceed those expected for age and educational background but do not meet the diagnostic criteria for dementia [1,2]. This condition involves measurable impairments in one or more cognitive domains, including memory, language, attention, executive function, social cognition, or visuospatial abilities, while functional independence in daily life is largely preserved. MCI can be classified into subtypes—amnestic or non-amnestic, single-domain or multidomain—based on the pattern of cognitive involvement. Among these, the amnestic subtype, characterized predominantly by memory impairment, is particularly associated with an increased risk of progression to Alzheimer’s disease dementia (AD) [3].

**Figure 1:**
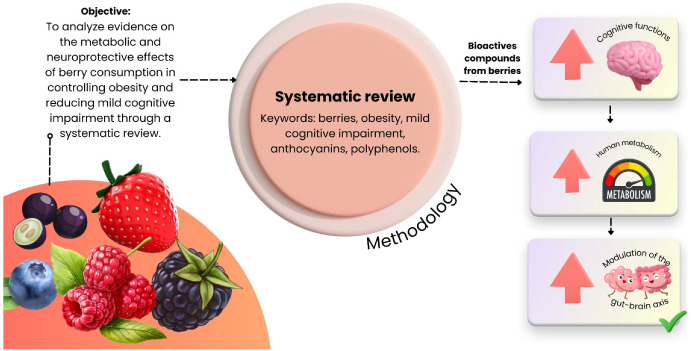
Neuroprotective effects from berries. Schematic illustration summarizing the main neuroprotective mechanisms associated with berry consumption. The figure was created by the authors using Canva (Canva Pty Ltd., Sydney, Australia, 2025, accessed on 12 November 2025, from https://www.canva.com).

The clinical course of MCI is heterogeneous: some individuals may revert to normal cognitive function, others remain cognitively stable over time, and a proportion progress to dementia. The risk of progression is significantly higher in individuals diagnosed with MCI than in age-matched cognitively unimpaired populations. As a result, differentiating MCI from both normal age-related cognitive changes and established dementia remains challenging, complicating accurate diagnosis and the interpretation of its clinical significance [4]. In this review, we focus on studies investigating the potential of berry-derived bioactive compounds to delay cognitive decline, with particular emphasis on the interactions among cognition, obesity, metabolic dysregulation, and inflammatory pathways (Figure 1).

### 1.1. Mild Cognitive Impairment (MCI)

Cognition comprises multiple mental abilities affected by aging and environmental factors, including memory, attention, language, processing speed, and executive function. While some domains, such as vocabulary, are preserved or may improve, others—particularly memory, abstract reasoning, and processing speed—decline with age [5]. Importantly, cognition extends beyond memory alone, involving a broad range of interrelated cognitive processes.

**Figure 2:**
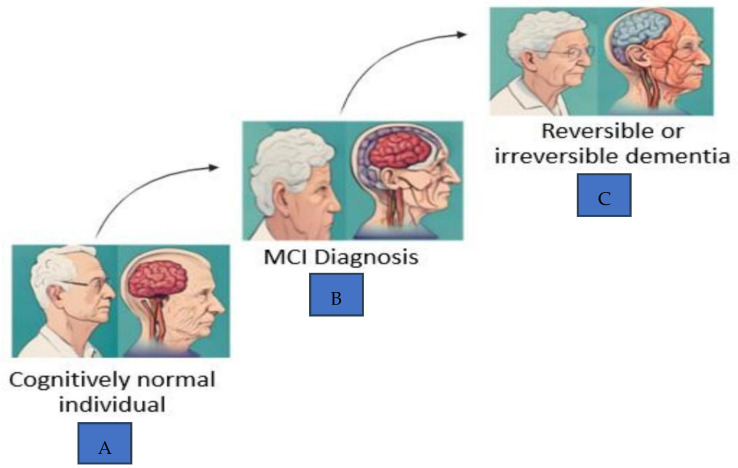
Evolution of MCI to dementia. AI-generated authorship with adaptations. Canva Platform, on 24 September 2024. (**A**) The person presents behavioral assessment scores within the normal range and without self-description of subjective cognitive decline. (**B**) A person diagnosed with MCI presents cognitive changes that influence daily life activities and functionality. (**C**) A person with dementia diagnosed through medical evaluation, presenting impairment of executive and higher cortical functions, with progressive MCI. MCI: mild cognitive impairment.

A 2024 study of over 26,000 older adults identified five data-driven MCI subtypes, with mixed MCI showing the highest risk of progression to dementia. These empirical classifications outperformed traditional diagnostic approaches in predicting Alzheimer’s disease [6]. Longitudinal evidence indicates that the annual conversion rate from MCI to dementia ranges from approximately 5% to 17%, and as global life expectancy continues to increase, the incidence of dementia is rising substantially [7]. Figure 2 illustrates the trajectory from MCI diagnosis to cognitive decline and the associated risk of dementia.

Characterized by subjective cognitive complaints, objective cognitive deficits, and preserved daily functioning, mild cognitive impairment is diagnosed through clinical evaluation and standardized instruments. Although multiple diagnostic criteria exist, they differ in their emphasis on memory versus multidomain impairment and on the source of subjective complaints. This concept is central to understanding neurodegenerative disease progression and identifying individuals at high risk for dementia and candidates for intervention [8]. Subtyping of MCI based on empirical data has clarified its clinical heterogeneity by identifying distinct forms, including amnestic and mixed MCI–severe, each associated with different risks of progression to dementia [9].

Mild cognitive impairment (MCI) is a pathological stage of cognitive decline beyond normal aging and is considered a distinct diagnostic entity. Original criteria emphasized subjective memory complaints, objective memory impairment, preserved global cognition and daily functioning, and absence of dementia. These criteria reflect the view of MCI as a prodromal phase of Alzheimer’s disease marked by early memory decline [10].

Recognized as an intermediate clinical stage, mild cognitive impairment is characterized by cognitive decline greater than expected for age while daily functioning remains largely preserved, and it often represents a prodromal phase of Alzheimer’s disease (AD). Approximately 40–75% of cases progress to AD, with annual conversion rates of 10–15%, although some individuals remain stable or revert to normal cognition. Positioned between healthy aging and dementia, this stage offers a critical window for early intervention and prevention strategies [11,12,13,14].

Individuals with MCI frequently exhibit neuropathological changes that resemble those observed in Alzheimer’s disease (AD) and related dementias, although typically to a lesser extent. Evidence from postmortem analyses in this population has demonstrated characteristic alterations consistent with early neurodegenerative processes, such as hallmark AD-related pathological features [15]:

- Aggregates of β-amyloid protein in the form of extracellular plaques and abnormal intracellular accumulations of hyperphosphorylated TAU protein, resulting in neurofibrillary tangles—hallmark pathological features of Alzheimer’s disease.
- Microscopic inclusions known as Lewy bodies, which are classically associated with Parkinson’s disease and dementia with Lewy bodies and are also observed in a subset of Alzheimer’s disease cases.
- Cerebrovascular alterations, including small infarcts and reduced cerebral perfusion, underscoring the contribution of vascular pathology to cognitive decline in MCI.

Neuroimaging investigations have further identified structural and functional alterations associated with mild cognitive impairment (MCI), including:

- Reduced hippocampal volume, a structure critically involved in memory formation and consolidation.
- Enlargement of the cerebral ventricles, reflecting progressive brain atrophy.
- Reduced cerebral glucose metabolism in specific cortical regions, indicating decreased availability and utilization of the brain’s primary energy substrate.

The Diagnostic and Statistical Manual of Mental Disorders, Fifth Edition (DSM-5), classifies dementia under the term Major Neurocognitive Disorder, which is characterized by a significant decline in memory and other cognitive domains that is sufficient to compromise independence in activities of daily living. The condition is marked by progressive cognitive deterioration, and affected individuals commonly present memory impairment with limited awareness of their deficits [16].

The development of mild cognitive impairment (MCI) is influenced by both non-modifiable factors, such as age and genetic predisposition, and modifiable factors, including low education, sedentary lifestyle, poor nutrition, alcohol use, socioeconomic disadvantages, and limited social engagement. Comorbid conditions—particularly metabolic, cardiovascular, neurological, and psychiatric disorders—along with brain injury, chronic inflammation, medication exposure, and genetic markers such as APOE polymorphisms, also contribute to MCI risk [17].

Recognizing age-related cognitive changes is essential for distinguishing normal aging from pathological decline and identifying opportunities for prevention and intervention. Even in the absence of dementia or MCI, subtle cognitive changes may occur with aging and should be considered when differentiating physiological from pathological processes [5].

### 1.2. Obesity and MCI

The World Obesity Atlas 2024 highlights obesity as a global public health challenge with substantial health consequences across all regions, particularly in low- and middle-income countries (LMICs). Currently, approximately 79% of adults and 88% of children living with overweight or obesity reside in LMICs, where the burden of obesity-related diseases is disproportionately high. In these settings, obesity is a major driver of premature disability and mortality due to its strong associations with type 2 diabetes, cardiovascular disease, and several cancers. The report further indicates that 78% of global deaths and 80% of disability-adjusted life years (DALYs) attributable to elevated body mass index occur in LMICs, compared with 22% and 20%, respectively, in high-income countries. These disparities underscore critical gaps in healthcare infrastructure, preventive strategies, and access to adequate nutrition. Moreover, the Atlas links rising obesity prevalence to broader environmental and societal pressures, emphasizing the roles of rapid economic development, urbanization, and environmental degradation. Collectively, these findings reinforce that obesity extends beyond an individual health issue, representing a systemic challenge shaped by socioeconomic and environmental determinants and requiring integrated, multisectoral responses encompassing public health policy, food system reform, and healthcare system strengthening [18].

Obesity is defined as the excessive accumulation of body fat, which may occur with or without alterations in fat distribution or adipose tissue function. Its etiology is complex and multifactorial, involving biological, environmental, and behavioral determinants that remain only partially understood. When excess adiposity results in systemic metabolic and inflammatory consequences, the condition is classified as clinical obesity—a chronic disease that impairs the normal functioning of organs, tissues, and overall physiological homeostasis.

Preclinical obesity refers to a stage characterized by excess adiposity in the absence of overt tissue or organ dysfunction, while conferring an increased risk of progression to clinical obesity [19].

The association between obesity and cognitive function has been increasingly documented in epidemiological studies. Yuan et al. [20] reported a prevalence of 18.5% for the association between obesity and mild cognitive impairment (MCI), with a significantly elevated risk observed only among men aged 75 years or older with high body mass index (BMI). No clear association was identified in men aged 60–75 years or in women over 60 years of age. Similarly, Quaye [21], in a large cohort study involving nearly 29,000 participants, demonstrated that obesity was associated with lower baseline cognitive performance; however, the effect of obesity on cognitive decline was attenuated after adjustment for cardiometabolic factors, including blood pressure and fasting glucose levels.

Despite obesity being a global public health challenge affecting approximately 38% of adults and nearly one in five children and adolescents, dietary imbalance remains an equally critical concern. A substantial proportion of the population follows dietary patterns that are low in antioxidants, polyphenols, and omega-3 fatty acids—nutrients that play essential roles in maintaining optimal brain function. Deficiencies in these dietary components may increase vulnerability to mood disorders and cognitive decline across the lifespan [22]. Evidence further suggests that interventions targeting obesity, including bariatric surgery and well-structured nutritional strategies, can lead to improvements in cognitive outcomes and reduce the risk of developing neurodegenerative diseases. Accordingly, effective management of obesity and its associated metabolic disturbances, particularly during midlife, may represent a valuable strategy for reducing the risk or delaying the onset of cognitive decline and dementia [23].

#### Obesity and Damage to Cognitive Functions

**Figure 3:**
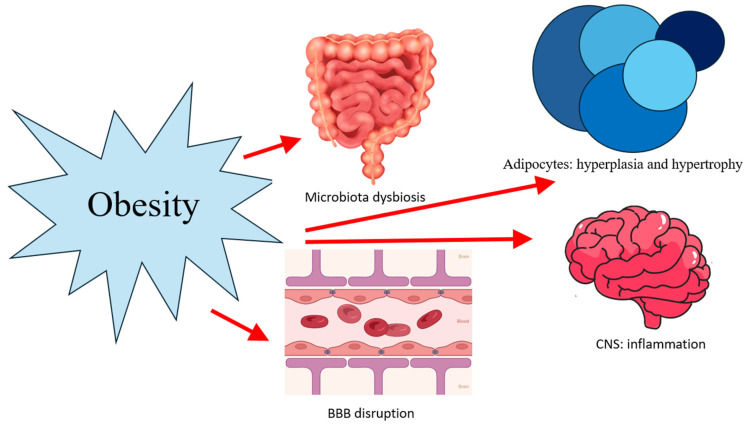
Effects of obesity on neurodegenerative diseases. Author himself. BBB: blood–brain barrier; CNS: central nervous system.

Obesity exerts systemic effects on multiple organs and is a major contributor to overall health deterioration. Within the central nervous system, excess adiposity has been associated with a range of homeostatic disruptions, including increased oxidative stress, neuroinflammation, protein misfolding and aggregation, mitochondrial dysfunction, hormonal dysregulation, insulin resistance, and compromised integrity of the blood–brain barrier (BBB) [24]. Collectively, these mechanisms—together with alterations in the gut–brain axis—underscore the multifaceted impact of obesity on neural function, as illustrated in Figure 3.

Importantly, obesity that develops early in life—particularly diet-induced obesity (DIO)—can exert long-lasting effects through the reprogramming of the innate immune system, even after metabolic abnormalities are corrected. One illustrative mechanism involves stearic acid, which, through activation of Toll-like receptor 4 (TLR4), induces chromatin remodeling that increases the accessibility of activator protein-1 (AP-1) binding sites. This epigenetic reprogramming shifts myeloid cell energy metabolism from oxidative phosphorylation toward glycolysis, thereby promoting the enhanced production and release of pro-inflammatory cytokines [25].

The alterations described above, together with the recognition of obesity as a state of chronic low-grade inflammation, establish a strong association between obesity and neurodegenerative disorders, including Alzheimer’s disease (AD), multiple sclerosis (MS), and Parkinson’s disease (PD).

The inflammatory state associated with obesity is largely driven by dysfunctional adipose tissue, which disrupts the secretion of cytokines and adipokines and promotes the recruitment of immune cells, such as macrophages and lymphocytes. In this context, insulin-resistant adipocytes release increased levels of circulating free fatty acids, which can activate Toll-like receptor 4 (TLR4) signaling in immune cells, including B lymphocytes. Activation of this pathway triggers the nuclear translocation of nuclear factor kappa B (NF-κB), a key transcription factor that amplifies the production of pro-inflammatory mediators, such as tumor necrosis factor-alpha (TNF-α) and interleukin-6 (IL-6) [26], while concurrently downregulating the expression of anti-inflammatory adipokines, including adiponectin [27].

A potential explanation for reduced adiponectin levels in obesity involves epigenetic mechanisms. Specifically, hypermethylation of promoter regions within the adiponectin gene, including the R2 site, mediated by enzymes such as DNA methyltransferase 1 (DNMT1), promotes heterochromatin formation. This epigenetic modification suppresses adiponectin gene transcription, thereby contributing to decreased adiponectin expression in obesity [28].

Adipsin, also known as complement factor D, plays a pivotal role at the interface of immune regulation, adipose tissue biology, and metabolic homeostasis. In addition to initiating the alternative complement pathway, adipsin contributes to adipogenesis and supports pancreatic β-cell function and insulin secretion. Elevated circulating adipsin levels have been associated with obesity, vascular dysfunction, and cardiovascular complications; however, accumulating evidence also indicates protective effects on β-cell survival and glycemic regulation. This apparent duality underscores the relevance of adipsin as both a potential biomarker and a therapeutic target. Importantly, its context-dependent effects must be carefully considered when evaluating adipsin-targeted interventions. Modulation of adipsin-related pathways may represent a novel strategy for cardiometabolic disease management, although further mechanistic and long-term studies are required to clarify its safety, efficacy, and clinical applicability. Collectively, adipsin emerges as a key adipokine with significant translational potential in metabolic and cardiovascular medicine [29].

Analogous to the role of adipsin in metabolic regulation, bioactive dietary compounds—particularly polyphenols—exert significant effects on both metabolic and brain function. These effects are mediated through interactions with key cellular and molecular pathways within the central nervous system (CNS). Importantly, the neuroprotective potential of polyphenols depends on their ability to cross the blood–brain barrier and accumulate in brain tissue, where they can support neuroplasticity and contribute to cognitive enhancement [30].

BBB disruption: Obesity-driven neuroinflammation can compromise the integrity of the blood–brain barrier (BBB), facilitating the infiltration of peripheral immune cells into the brain and promoting microglial activation. This inflammatory cascade amplifies immune responses within the central nervous system (CNS) and has been associated with an increased risk of mild cognitive impairment (MCI) [31].

Adipocytes: hyperplasia and hypertrophy: Adipose tissue has been recognized as a source of neurotrophic factors, including nerve growth factor (NGF) and brain-derived neurotrophic factor (BDNF), both of which play important roles in the regulation of metabolic activity and immune function. In obesity, alterations in the expression and signaling of these molecules have been reported, along with hyperleptinemia and an increased density of mast cells within subcutaneous abdominal adipose depots [32]. Under physiological conditions, adipose tissue supports essential biological processes through tightly regulated autocrine, paracrine, and endocrine signaling pathways [33].

Obesity induces a persistent, low-grade inflammatory state driven by chronic expansion and dysfunction of adipose tissue, which in turn promotes widespread metabolic disruption. Hypertrophic adipocytes and infiltrating immune cells—particularly macrophages—secrete pro-inflammatory mediators such as tumor necrosis factor-alpha (TNF-α), interleukin-6 (IL-6), and interleukin-1 beta (IL-1β), thereby sustaining systemic inflammation. These cytokines impair insulin signaling through activation of pathways including nuclear factor kappa B (NF-κB) and c-Jun N-terminal kinase (JNK), contributing to the development of insulin resistance and type 2 diabetes. This chronic inflammatory environment is further characterized by dysregulated adipokine release, which mediates both the initiation of inflammation and the progression of insulin resistance, thereby linking excess adiposity to metabolic dysfunction [34]. Collectively, these mechanisms underscore meta-inflammation as a critical therapeutic target, and interventions aimed at modulating adipose tissue–driven inflammation—through lifestyle, nutritional, or pharmacological strategies—may substantially mitigate obesity-related metabolic risk, particularly in the context of the rising global prevalence of obesity [35]

Central nervous system inflammation: Although the central nervous system is a highly regulated environment primarily protected by the blood–brain barrier (BBB), accumulating evidence indicates that this structure is dynamic rather than static. The BBB actively regulates the transport of ions and nutrients, restricts exposure to circulating toxins, and controls immune cell trafficking, thereby maintaining cerebral homeostasis. However, BBB integrity is susceptible to disruption in a range of neurological conditions, including neurodegenerative disorders and stroke, where barrier dysfunction can exacerbate disease pathology. Understanding BBB heterogeneity and context-dependent alterations is therefore critical, as these features influence disease progression and contribute to the limited efficacy of many neurological therapeutics. Moving beyond the simplistic dichotomy of an “open versus closed” BBB toward a more nuanced, functional model is essential for advancing the development of accurate diagnostic tools and targeted therapeutic strategies aimed at restoring or modulating barrier function [36].

Chronic low-grade inflammation associated with obesity establishes a sustained pro-inflammatory milieu that increases susceptibility to long-term metabolic disorders, particularly type 2 diabetes. In this context, dysfunctional adipose tissue releases elevated levels of pro-inflammatory cytokines, such as tumor necrosis factor-alpha (TNF-α) and interleukin-6 (IL-6), which disrupt insulin signaling pathways and contribute to the development of insulin resistance. These inflammatory mediators also impair pancreatic β-cell function, leading to reduced insulin secretion and accelerating diabetes progression. Consequently, obesity-related inflammation represents a critical mechanistic link between excess adiposity and metabolic dysregulation. Elucidating this pathway is essential, as it highlights potential therapeutic targets to disrupt the obesity–diabetes axis. Interventions aimed at reducing systemic inflammation—through lifestyle modification or pharmacological strategies—may therefore attenuate diabetes risk even in the absence of substantial weight loss, underscoring the importance of targeting inflammation in the prevention or delay of type 2 diabetes onset [37,38].

Microglial cells play a central role in neurodegenerative processes by releasing pro-inflammatory cytokines, generating excitotoxic mediators such as glutamate, and activating enzymatic systems, including NADPH oxidase. These mechanisms contribute to neuronal injury and apoptosis. For example, interleukin-1 beta (IL-1β) released by activated microglia amplifies neuroinflammation by stimulating neighboring microglia and astrocytes, which subsequently produce additional cytotoxic and pro-inflammatory mediators [39].

Another concept closely associated with obesity is meta-inflammation, a state of chronic, low-grade inflammation characterized by elevated circulating levels of pro-inflammatory markers, including interleukin-6 (IL-6), C-reactive protein (CRP), and tumor necrosis factor (TNF). Gut microbiota dysbiosis—defined as an imbalance in microbial composition—may further exacerbate this condition by promoting systemic inflammatory responses. In this context, dietary strategies incorporating anthocyanin-rich foods, such as cherries, raspberries, black soybeans, blueberries, strawberries, and plums, have been proposed as potential modulators of meta-inflammation. A key advantage of these bioactive compounds is their favorable safety profile, as they are generally well tolerated and lack the significant adverse effects commonly associated with pharmacological interventions [40].

Gut–brain axis: Emerging evidence highlights the influence of the gut microbiota on central nervous system (CNS) function and, conversely, the ability of the CNS to modulate intestinal microbial composition—a dynamic interaction referred to as the gut–brain axis. This bidirectional communication involves multiple pathways, including the central, enteric, and autonomic nervous systems, as well as the hypothalamic–pituitary–adrenal (HPA) axis [41]. Experimental studies in rodents fed high-fat diets have demonstrated marked alterations in gut microbial diversity, accompanied by impaired synaptic plasticity, deficits in exploratory behavior and cognitive performance, and increased vulnerability to anxiety-like behaviors [42].

The gut–brain axis plays a critical role in the regulation of cognitive functions, including memory, perception, and attention. Intestinal microorganisms can produce neurotransmitters and bioactive signaling molecules that influence not only brain development but also ongoing neural activity and cognitive processes [41].

During neuroinflammatory states, disruptions of the gut microbial ecosystem—commonly referred to as dysbiosis—may trigger inflammatory signaling within the brain, thereby compromising cognitive performance [43]. The gut microbiota also plays a regulatory role in modulating the hypothalamic–pituitary–adrenal (HPA) axis, a central component of stress responsiveness and cognitive regulation. Dysbiosis has been shown to increase HPA axis reactivity, which can negatively affect learning and memory processes. In this context, nutritional strategies, including diets rich in berries or berry-derived supplements, have demonstrated potential to favorably modulate gut microbiota composition, with downstream benefits for cognitive function [44].

Recent evidence indicates that alterations in gut microbiota composition and function may contribute to the progression of Alzheimer’s disease (AD) by modulating neuroinflammatory processes, promoting amyloid-beta deposition, and influencing TAU-related pathology [45]. The gut microbiome synthesizes a wide range of neuroactive metabolites and neurotransmitters that play critical roles in regulating neurochemical signaling and behavioral outcomes. Notably, aromatic amino acids such as tryptophan, tyrosine, and phenylalanine—precursors of serotonin, dopamine, and norepinephrine—are metabolized through microbial enzymatic pathways. These metabolites can influence central nervous system activity primarily via vagal afferent signaling, thereby establishing bidirectional communication between the intestinal environment and brain function [46]. In addition, compromised intestinal barrier integrity may permit the translocation of microbial-derived toxins and pro-inflammatory molecules into systemic circulation, contributing to systemic and neuroinflammation and exacerbating neuronal injury [47].

Intestinal dysbiosis can promote the release of pro-inflammatory mediators, including cytokines such as tumor necrosis factor-alpha (TNF-α), interferon-gamma (IFN-γ), various interleukins (ILs), and lipopolysaccharides (LPSs). These factors contribute to increased intestinal permeability, commonly referred to as “leaky gut.” The resulting barrier dysfunction facilitates the translocation of inflammatory mediators into systemic circulation and the enteric nervous system, with subsequent propagation to the peripheral nervous system (PNS) and ultimately the central nervous system (CNS). This inflammatory cascade promotes chronic neuroinflammation and compromises the integrity of the blood–brain barrier (BBB) [48].

### 1.3. Bioactive Compounds and Berries

A wide range of health-promoting effects has been attributed to fruits rich in bioactive phytochemicals, including flavonoids, phenolic acids, tannins, organic acids, tocopherols, dietary fiber, as well as essential vitamins and minerals. The concentration and distribution of these compounds vary considerably across different berry species. Due to their seasonal availability, these fruits are commonly processed into various products, such as juices, jams, jellies, purées, and ice creams [49].

Berries are rich sources of dietary fiber, vitamins, minerals, and a wide array of phytochemicals. Their predominant bioactive constituents are phenolic compounds, particularly flavonoids—including anthocyanins, flavonols, flavones, flavanones, and isoflavonoids—as well as tannins and phenolic acids. Owing to their high polyphenol content, berries have been extensively investigated for their potential roles in the prevention and management of chronic diseases, largely through antioxidant and anti-inflammatory mechanisms. These effects are especially relevant to conditions in which oxidative stress and inflammation are central pathogenic features, such as diabetes, cardiovascular disease, and neurodegenerative disorders [50]. Although this review focuses on berries, other fruit-derived sources, such as grape extracts, are briefly considered because of their comparable polyphenolic profiles. These foods share key bioactive compounds, including anthocyanins and other flavonoids, which exert similar biological effects on metabolic and neural pathways. Recognizing these analogues provides important context for interpreting berry-specific effects within the broader framework of dietary polyphenols and supports a more comprehensive understanding of how fruit-derived bioactives may influence obesity and cognitive function.

Among dietary sources of bioactive compounds (BACs), berries are considered particularly valuable. Species belonging to the *Rosaceae* family (e.g., strawberries, raspberries, and blackberries) and the *Ericaceae* family (e.g., blueberries and cranberries) are especially rich in phenolic acids, flavonoids—including anthocyanins and flavonols—tannins, and vitamin C. These constituents may act synergistically or independently to confer health benefits, such as attenuation of inflammation, cardiovascular protection, and a reduced risk of certain cancers [51].

Preclinical evidence indicates that flavonoids exert neuroprotective effects by modulating learning and memory processes. Experimental studies suggest that these compounds may attenuate age-related cognitive decline, reduce neuronal apoptosis, and confer protection against ischemic and neurodegenerative damage, thereby contributing to improved brain function [52].

Polyphenols are bioactive molecules widely recognized for their potent antioxidant properties, with flavonoids representing the most extensively studied subclass [53]. Based on their chemical structure, polyphenols are commonly classified into four major groups: flavonoids, phenolic acids, stilbenes, and lignans. Curcuminoids, such as curcumin, are frequently discussed alongside polyphenols due to their overlapping biological activities, although they do not strictly fall within these structural categories [54].

The review by Carrillo [55] highlights the growing body of evidence indicating that polyphenols—particularly those derived from berries—play an important role in supporting cognitive health during aging. Beyond their antioxidant properties, these compounds modulate key molecular pathways involved in synaptic plasticity and memory formation, including brain-derived neurotrophic factor (BDNF) and cAMP response element-binding protein (CREB). Clinical evidence suggests that polyphenol supplementation may improve specific cognitive domains, such as executive function and episodic memory. Importantly, several polyphenols are capable of crossing the blood–brain barrier, enabling direct neuroprotective effects within the central nervous system. The authors further note that regular dietary intake of berry-derived polyphenols may contribute to delaying age-related cognitive decline. However, heterogeneity in study design, dosing regimens, and participant characteristics remains a significant limitation. The standardization of nutraceutical formulations will therefore be critical to improve reproducibility across studies. Overall, polyphenol-rich interventions emerge as a promising, safe, and accessible strategy, although large-scale, long-term clinical trials are still required to confirm efficacy and establish evidence-based guidelines for cognitive health promotion.

Epicatechin (EC), a flavonoid abundant in foods such as cocoa, tea, and berries, has received considerable attention for its biological properties [56]. This compound is suggested to exert neurovascular protective effects by modulating redox homeostasis, reducing oxidative stress, and promoting vascular health, which collectively may support cognitive performance [57].

Blueberries are a rich source of polyphenols and have been associated with potential benefits for cognitive performance and mood regulation. A systematic review including eleven studies reported that eight demonstrated improvements in cognitive outcomes following blueberry consumption or supplementation, particularly in domains such as short-term memory, long-term memory, and spatial memory. With respect to mood-related outcomes, one trial reported a significant improvement in positive affect, whereas four studies did not observe meaningful changes. Although these findings suggest potential cognitive benefits, substantial heterogeneity in study design, supplementation regimens, and anthocyanin content limits direct comparisons across studies and precludes definitive conclusions [50].

A 12-week strawberry supplementation trial conducted in overweight, middle-aged adults with insulin resistance highlighted potential cognitive and emotional benefits associated with berry intake. Participants exhibited improvements in measures of memory interference, suggesting enhanced executive control of information processing. In addition, reductions in depressive symptoms were observed, indicating possible mood-regulating effects. Although metabolic parameters were also assessed, changes in cognitive and affective outcomes were the most consistent findings. These results suggest that strawberry consumption may confer neuroprotective support in populations at increased risk for metabolic and cognitive decline. The concurrent effects on memory and mood underscore the broader influence of dietary polyphenols on brain function. However, modest sample size and relatively short intervention duration limit the generalizability of these findings. Larger, longer-term trials are therefore warranted to establish the durability and clinical relevance of these effects [58].

Results from a 28-week Randomized Controlled Crossover Trial in adults with prediabetes demonstrated that a feasible dietary intake of 2.5 servings of strawberries per day may contribute to metabolic health in adults with prediabetes. The study states that future research should evaluate whether lower doses provide clinical benefits, as well as whether strawberry supplementation improves glycemic control and slows disease progression in prediabetes and type 2 diabetes, in conjunction with pharmacological interventions and lifestyle changes. Therefore, considering that strawberries significantly improved antioxidant markers, fasting blood glucose, and inflammation in individuals with prediabetes, strawberry consumption can be recommended in nutritional therapy as a practical, non-pharmacological intervention for both the management of prediabetes and the prevention of type 2 diabetes [59].

Regular blueberry consumption may be associated with benefits in episodic memory among older adults with MCI and subjective cognitive decline, as well as improvements in language performance in individuals with MCI. However, these observations require confirmation through larger, multicenter studies to establish their generalizability and long-term effects [60].

The therapeutic interest in berries more broadly arises from their high polyphenol content, which contributes to the modulation of oxidative stress and inflammatory pathways—key mechanisms implicated in the pathophysiology of diabetes, cardiovascular disease, cancer, and neurodegenerative disorders [61]. Among berries, blueberries have received particular attention due to their pronounced antioxidant and anti-inflammatory properties, as well as their potential neurocognitive benefits. Clinical evidence indicates that older adults with early memory decline experienced improvements in memory performance following regular consumption of blueberry juice [62].

A randomized controlled trial indicated that cranberry supplementation over a 12-week period may contribute to healthier cognitive aging. Older adults receiving the intervention demonstrated improvements in episodic memory, a cognitive domain commonly affected by advancing age. Neuroimaging assessments revealed increased cerebral perfusion, suggesting that cranberries may enhance vascular pathways that support cognitive function. In addition to these neurological effects, participants exhibited reductions in low-density lipoprotein (LDL) cholesterol, reinforcing the cardioprotective potential of berry-derived polyphenols. Body weight was monitored to provide a metabolic context and to ensure that observed effects were not confounded by changes in adiposity. Collectively, these findings suggest that cranberries may confer concurrent benefits for brain and cardiovascular health. The integration of cognitive and cardiometabolic outcomes highlights their potential as a multifaceted dietary strategy. Nevertheless, the modest sample size and limited intervention duration warrant cautious interpretation. Further studies with larger cohorts, longer follow-up periods, and mechanistic exploration are needed to define optimal dosing and substantiate clinical applicability. Overall, cranberries emerge as a safe and promising nutritional approach to support memory and vascular function in older adults [63].

Acute administration of haskap berry extract in older adults was associated with measurable cognitive and vascular benefits. Participants exhibited improvements in episodic memory performance, suggesting short-term enhancement of memory-related processes. In addition, reductions in blood pressure were observed, indicating favorable effects on vascular function. Collectively, these findings underscore the potential of haskap berries as a dietary strategy to concurrently support cognitive health and cardiovascular regulation in aging populations [64].

Black raspberries are a notable source of bioactive phenolic compounds, particularly ellagic acid and anthocyanins, which have been validated in clinical settings for their chemopreventive effects against carcinogenesis [65]. Similarly, evidence from both preclinical and clinical studies indicates that strawberries possess pronounced antioxidant and anti-inflammatory properties. These effects are largely mediated by their complex matrix of vitamins, polyphenolic compounds, and secondary metabolites, which collectively modulate redox homeostasis and inflammatory signaling pathways [66]. In addition, blackcurrant powder has been shown to reduce the activity of specific biomarkers associated with colon cancer, partly through prebiotic mechanisms [67].

According to Tandoro [68], supplementation with black raspberry in overweight older adults with mild dementia was associated with measurable cognitive benefits, as reflected by lower scores on the Clinical Dementia Rating (CDR) scale. The intervention was also linked to reductions in body mass index (BMI) and decreased circulating levels of inflammatory proteins implicated in dementia progression. These findings suggest that bioactive constituents of black raspberry may exert combined neuroprotective and metabolic effects. Collectively, the results support the concept that berry-based dietary strategies may represent a multifaceted intervention for populations at increased metabolic and cognitive risk. Nevertheless, the authors emphasize that longer-duration and larger-scale clinical trials are required to confirm the durability and clinical relevance of these effects.

While dietary patterns rich in saturated fats and simple sugars are known to impair insulin signaling and negatively affect cognitive processes, antioxidant components such as phenolic compounds and dietary fiber have been shown to improve insulin sensitivity and support neurogenesis [69]. In particular, flavonoids exhibit protective properties that may counteract oxidative stress, excessive weight gain, insulin resistance, inflammation, and cognitive decline [70].

Epidemiological evidence further associates the consumption of polyphenol-rich red fruits with a reduced risk of cancer, cardiovascular disease, Alzheimer’s disease (AD), and other chronic conditions [71]. These fruits, particularly berries, are rich in anthocyanins and other antioxidant compounds that may support brain function by protecting neurons and enhancing memory-related processes [72]. In addition, their dietary fiber content contributes to appetite regulation and weight management, thereby reducing the risk of obesity and obesity-related complications [73].

Experimental studies provide important mechanistic insights into the neuroprotective actions of phenolic compounds. Supplementation with purified phenolics has been shown to protect mice against TAU hyperphosphorylation and cognitive impairment induced by diabetes in the streptozotocin model [74] or by high-fat diet exposure [75]. Notably, these protective effects were not observed in lean, non-diabetic animals, suggesting that phenolic compounds primarily exert protective rather than enhancing actions and are particularly effective under conditions of metabolic stress. Consistent with this concept, intake of jaboticaba peel extract (MJP) prevented high-fat diet–induced TAU phosphorylation through attenuation of adiposity and peripheral insulin resistance, as well as through modulation of insulin signaling via inhibition of glycogen synthase kinase-3 (GSK-3). Additional evidence indicates a direct role of MJP in improving hepatic insulin sensitivity through activation of the insulin receptor substrate–AKT–forkhead box O1 (IRS–AKT–FoxO1) signaling pathway, independent of changes in body weight [76].

Among the bioactive constituents identified in jabuticaba peel extract (MJP), cyanidin-3-O-glucoside, ellagic acid, and carotenoids are particularly noteworthy due to their ability to cross the blood–brain barrier and reach the brain parenchyma [77]. Once within the central nervous system, these compounds interact with neuronal receptors, kinases, transcription factors, neurotrophins, and enzymes involved in antioxidant defense, inflammatory regulation, and insulin signaling pathways [69]. Experimental evidence indicates that the activities of key antioxidant enzymes—such as superoxide dismutase (SOD), catalase (CAT), and glutathione peroxidase (GPx)—are increased in the frontal lobe, suggesting attenuation of oxidative stress through activation of endogenous protective mechanisms [78]. In addition, consumption of jabuticaba juice has been associated with prevention of high-fat diet–induced TAU protein phosphorylation, either indirectly through reductions in adiposity and peripheral insulin resistance or directly via modulation of insulin signaling through inhibition of glycogen synthase kinase-3 (GSK-3). Proper insulin signaling appears to be central to this effect, as phosphorylation of insulin receptor substrate (IRS) on tyrosine residues activates protein kinase B (AKT), which subsequently inhibits GSK-3 activity, thereby preventing TAU phosphorylation [76,78].

In contrast to the detrimental effects of dietary saturated fats and refined sugars on insulin sensitivity and cognitive function, antioxidant dietary components such as phenolic compounds and dietary fiber have been shown to improve metabolic regulation and support neurogenesis [69]. Flavonoids, in particular, exhibit strong potential to mitigate oxidative stress, excessive weight gain, insulin resistance, inflammation, and cognitive impairments [70]. These phytochemical constituents are widely distributed in plant-based foods, with especially high concentrations found in berries, grape-derived products, and solanaceous fruits such as tomatoes [79].

Quercetin, a widely studied flavonoid, exerts potent antioxidant and anti-inflammatory effects through modulation of key intracellular signaling pathways, notably nuclear factor erythroid 2–related factor 2 (Nrf2) and nuclear factor kappa B (NF-κB). Through activation of these pathways, quercetin enhances the expression of endogenous antioxidant enzymes while suppressing pro-inflammatory gene transcription, thereby mitigating oxidative stress and inflammatory responses in chronic disease contexts [80]. In neuronal cells, its principal mechanism involves suppression of reactive oxygen species (ROS) and inflammatory mediators via modulation of the Nrf2/heme oxygenase-1 (HO-1) signaling axis [81]. Quercetin is widely distributed in fruits and vegetables and has demonstrated neuroprotective properties by interfering with amyloid-beta (Aβ) aggregation, destabilizing fibril formation, and attenuating Aβ-induced neurotoxicity—processes relevant to Alzheimer’s disease (AD) pathophysiology [82]. In addition to its neuroprotective actions, clinical evidence indicates that quercetin supplementation confers metabolic benefits, including significant reductions in systolic blood pressure and fasting insulin levels, highlighting its potential to modulate cardiometabolic risk factors through systemic antioxidant and anti-inflammatory mechanisms. Although effects on other metabolic parameters remain inconsistent, these findings support the promise of quercetin in nutritional and therapeutic strategies aimed at mitigating inflammation-driven conditions. Further research is warranted to establish optimal dosing regimens and evaluate long-term clinical outcomes [83,84].

Despite the accumulating evidence supporting the biological effects of polyphenols, important knowledge gaps remain regarding their role in modulating obesity as a contributing factor to mild cognitive impairment (MCI). Current evidence highlights a complex, bidirectional relationship between body weight regulation and cognitive function, largely mediated by inflammatory pathways and cytokine signaling. Within this framework, the present study aimed to systematically review the available literature on the metabolic and neuroprotective effects of red fruit consumption, with a particular focus on obesity-related mechanisms and their potential implications for mitigating mild cognitive impairment.

## 2. Methods

This systematic review was conducted and reported in accordance with the Preferred Reporting Items for Systematic Reviews and Meta-Analyses (PRISMA) 2020 guidelines (Table S1). A comprehensive literature search was performed in PubMed, Scopus, and Web of Science, complemented by additional sources, covering the period from 1 April to 30 June 2025. The risk of bias of the included studies was assessed using validated tools appropriate to each study design. The search strategy incorporated the following key terms: mild cognitive impairment, cognitive decline, body weight, obesity, adiposity, and berries. These terms were searched both individually and in combination using Boolean operators to refine retrieval. Searches were conducted within article titles, abstracts, and keywords to ensure comprehensive coverage. No restrictions were applied with respect to language or journal impact factor, thereby maximizing the inclusivity and scope of the retrieved literature.

Table 1 describes Population, Intervention, Comparator, Outcomes and Type of Study (PICO) as a strategy to verify eligibility criteria.

**Figure 4:**
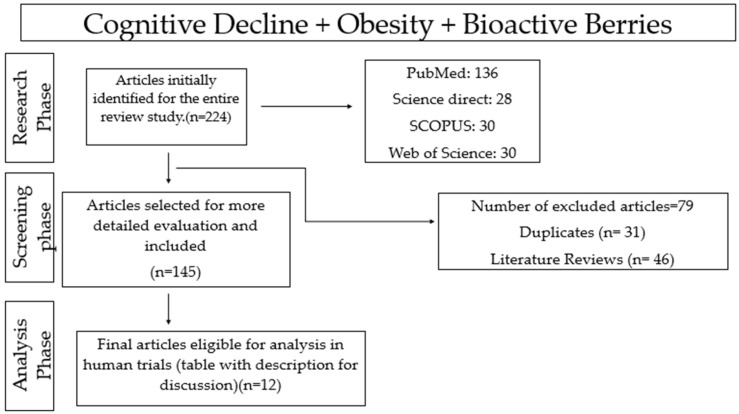
The flowchart refers to the method for selecting publications using scientific databases. The final “eligible articles” refers exclusively to articles selected for analysis in human trials.

Data collection was conducted between 1 April and 30 June 2025, with the final database search completed on 30 June 2025. An initial total of 224 records was identified based on the predefined search terms. After application of the inclusion and exclusion criteria, 145 studies were retained for full-text assessment. The screening and extraction of data were performed by only one reviewer, who was responsible for the analysis, thus avoiding possible disagreements. Eligible studies were required to address the triad of bioactive compounds, obesity, and cognitive outcomes through human clinical research. Studies conducted in laboratory animals were not included in the quantitative or qualitative synthesis but were occasionally referenced in the Discussion Section to provide mechanistic support. Review articles and duplicate records were excluded. Priority was given to studies published between 2004 and 2024, with approximately 50% of the included literature originating from the past decade, underscoring the contemporary relevance of the topic. Of the 145 full-text articles assessed, 12 studies met all eligibility criteria and were included in the final analysis, as they provided sufficient data to comprehensively evaluate the relationship between berry consumption, obesity management, and cognitive decline in human populations. The review protocol was not preregistered. The study selection process is summarized in the flow diagram presented in Figure 4.

## 3. Results

The evaluation of bioactive compound activity in humans was focused on studies that assessed therapeutic effects in the presence of objectively measured cognitive alterations, as determined by validated neuropsychological tests. Studies conducted exclusively in animal models were excluded from the final analysis, given the need to evaluate cognition as a human-specific outcome and a clinically relevant stage preceding dementia. In this context, dementia was considered a predominantly human neurodegenerative condition. Obesity was incorporated as a central variable based on evidence indicating that weight gain–related metabolic and inflammatory disturbances may contribute to neurodegenerative processes, including β-amyloid accumulation and TAU protein hyperphosphorylation.

Table 2 summarizes the studies identified through the search strategy that specifically evaluated the effects of bioactive compounds on cognitive outcomes. The table presents the investigated compounds, the corresponding berry sources, and the main reported findings, including cognitive and neurobehavioral assessments, relevant biological markers, and additional physiological parameters when available. The characteristics of the included studies are organized in chronological order of publication. The emphasis on cognitive outcomes reflects the primary objective of this review, which is to explore the potential role of bioactive compound consumption as a protective strategy against cognitive impairment. Although obesity was considered an important contextual factor, cognitive decline was not treated as an outcome exclusively dependent on obesity, as cognitive deficits may occur independently of weight status.

The findings of the included studies indicate that no single intervention is sufficient to prevent cognitive decline; rather, cognitive outcomes appear to be influenced by multiple modifiable lifestyle factors, particularly dietary patterns. Several studies highlighted associations between healthier diets—characterized by higher intake of fruits, vegetables, legumes, and whole grains—and better cognitive performance with aging. In addition, evidence from the analyzed trials supports a role for diet–gut–brain interactions in modulating cognitive function, suggesting that nutritional quality may contribute to the maintenance of cognitive health over time.

A study conducted by researchers at Brigham and Women’s Hospital reported that women who consumed two or more weekly servings of strawberries and blueberries exhibited a delay in memory decline of up to 2.5 years. In the same study, supplementation with wild blueberry extract at a dose of 222 mg was associated with reductions in both systolic and diastolic blood pressure [85].

Based on the evidence indicating potential cognitive benefits, the analysis focused on clinical trials conducted exclusively in human participants that evaluated berry consumption. Particular attention was given to participant characteristics, the specific bioactive compounds investigated, and their respective concentrations, as summarized in Table 1. The included studies varied in their intervention formats, with some administering berries in their whole, fresh form, while others provided isolated bioactive compounds in sachet or capsule formulations. All selected trials employed randomized, double-blind designs, with intervention durations ranging from acute exposures of several hours to longer-term protocols lasting several months.

**Table 2:** Characteristics of studies conducted in humans, with an impact on cognition, eligible for review.

Authors	Food Matrix Evaluated	Dose, Time	Study Design	Main Results (Biomarkers Used)
(Krikorian, R.; Skelton, M.R.; Summer, S.S.; Shidler, M.D.; Sullivan, P.G., 2022) [86]	Blueberry	0.5 to 1.0 c whole-fruit equivalent (Blueberry); 12 weeks. There is no description by the authors regarding the concentrations of Anthocyanins in each cup (c) of Blueberry; taken orally.	A total of 155 participants aged between 50 and 65 years were initially randomized; however, 27 individuals completed the study and were included in the final analysis. Eligibility criteria included a body mass index (BMI) ≥ 25 kg/m^2^, the presence of subjective cognitive complaints reflecting perceived decline from a previous level of cognitive functioning, the ability to understand and adhere to the study protocol, and provision of written informed consent.	Blueberry supplementation demonstrated neurocognitive benefits in middle-aged individuals with insulin resistance and an elevated risk of future dementia. After 12 weeks of intervention, lexical access performance significantly improved in the blueberry (BB) group (F(1,24) = 10.67, *p* = 0.003; Cohen’s f = 0.66). Perceived everyday memory difficulties, assessed using the Everyday Memory Questionnaire, were also significantly reduced in the BB group, particularly with respect to forgetting and encoding-related failures (F(1,24) = 4.93, *p* = 0.03; Cohen’s f = 0.45). In addition, fasting insulin levels were measured at baseline and after 12 weeks, revealing a significant reduction in the BB group following supplementation (F(1,24) = 4.62, *p* = 0.04; Cohen’s f = 0.44).
(Devore, E.E.; Kang, J.H.; Breteler, M.M.; Grodstein, F., 2012) [87]	Blueberry and Strawberry	145.4 to 684.1 mg/day of flavonoids (taken orally), without specifying which flavonoids were evaluated and the concentration of each of them. Foods were specified in a common unit or serving size = ½ cup blueberries. Frequency: ≥6 times per day.” Intakes of 31 individual flavonoids representing six major flavonoid subclasses (anthocyanidins, flavonols, flavones, flavanones, flavan-3-ols, and polymeric flavonoids) were also calculated. Time of study: 6 years.	A large human cohort study included 16,010 female participants aged over 70 years, with no history of stroke and a mean body mass index (BMI) of 26.0 kg/m^2^	Higher blueberry consumption was significantly associated with a slower rate of cognitive decline, including global cognitive scores (*p* for trend = 0.010), verbal cognition (*p* for trend = 0.016), and performance on the Telephone Interview for Cognitive Status (*p* for trend = 0.027). The mean difference in the rate of global cognitive decline during the follow-up period was 0.04 standard units (95% CI: 0.01–0.07) when comparing women who consumed ≥1 serving of blueberries per week with those who consumed <1 serving per month. These effect estimates were comparable to those reported in previous cohort studies and suggest that berry consumption may delay cognitive aging by up to 2.5 years. Furthermore, higher intakes of anthocyanins and total flavonoids were independently associated with slower rates of cognitive decline, providing additional support for the neuroprotective role of berry-derived polyphenols.
(Doraiswamy, P.M.; Miller, M.G.; Hellegers, C.A.; Nwosu, A.; Choe, J.; Murdoch, D.M., 2023) [88]	Blueberry	36 g per day of freeze-dried blueberry powder in a divided dose consumed with breakfast and dinner (taken orally), per 12 weeks. The author does not mention the bioactive compounds in blueberries and their respective concentrations in each dose administered during the experiment.	Participants received blueberry supplementation while abstaining from other anthocyanin-rich foods and beverages throughout the intervention period. The study enrolled 43 participants aged 55 to 85 years who were English-speaking, clinically stable, and met diagnostic criteria for amnestic mild cognitive impairment. These criteria included impaired delayed verbal recall with otherwise normal or near-normal global cognition and preserved functional abilities.	The biomarkers Aβ40, Aβ42, the Aβ42/Aβ40 ratio, phosphorylated tau at threonine 181 (pTAU181, pg/mL), the pTAU181/Aβ42 ratio, neurofilament light chain (NfL, pg/mL), glial fibrillary acidic protein (GFAP, pg/mL), and brain-derived neurotrophic factor (BDNF, pg/mL) were evaluated at baseline and after the intervention. No statistically significant changes were observed between time points. Baseline Aβ40 levels were 114.45 ± 4.67 pg/mL and remained stable after treatment (114.73 ± 4.27 pg/mL; *p* = 0.55). Similarly, Aβ42 concentrations showed no change from baseline (5.75 ± 0.42 pg/mL) to post-treatment (5.75 ± 0.36 pg/mL; *p* = 0.50). The Aβ42/Aβ40 ratio remained unchanged (0.050 ± 0.002 at both time points). For tau-related biomarkers, pTAU181 levels were 37.24 ± 3.31 pg/mL at baseline and 38.04 ± 2.58 pg/mL post-treatment (*p* = 0.64), while the pTAU181/Aβ42 ratio showed no significant difference between baseline (6.72 ± 0.65) and post-treatment (6.79 ± 0.56; *p* = 0.56). Neurodegeneration and glial activation markers also remained stable. NfL concentrations were 30.09 ± 4.64 pg/mL at baseline and 44.25 ± 16.85 pg/mL after treatment (*p* = 0.80), and GFAP levels were 239.86 ± 31.15 pg/mL at baseline compared with 234.27 ± 26.98 pg/mL post-treatment (*p* = 0.34). Likewise, BDNF concentrations did not differ significantly between baseline (1685.48 ± 480.43 pg/mL) and post-treatment (1850.15 ± 436.63 pg/mL; *p* = 0.61).
(Wood, E.; Hein, S.; Mesnage, R.; Fernandes, F.; Abhayaratne, N.; Xu, Y.; Zhang, Z.; Bell, L.; Williams, C.; Rodriguez-Mateos, A., 2023) [89]	Wild blueberry	26 g freeze-dried WBB powder (equivalent to 178 g fresh WBB) containing 302 mg anthocyanins), once daily, taken orally, 12 weeks.	A randomized, double-blind, parallel-group clinical design involving 61 healthy older adults of both sexes, aged 65–80 years, with a body mass index (BMI) ranging from 18 to 35 kg/m^2^. All participants were required to be capable of understanding the nature of the study and providing informed consent.	Wild blueberry (WBB) intervention improved specific aspects of cognitive function; however, no significant differences were observed for other measures of the Auditory Verbal Learning Test (AVLT). In the Task Switching Task (TST), 12 weeks of daily WBB supplementation resulted in a significant improvement in overall accuracy, corresponding to an 8.5% increase in performance compared with placebo (F(1,46) = 5.05, *p* = 0.029).
(Miller, M.G.; Hamilton, D.A.; Joseph, J.A.; Shukitt-Hale, B., 2018) [90]	Blueberry	Freeze dried blueberries (24 g/day); 90 days of this project. equivalent to 1 cup of fresh blueberries; This serving of blueberries contains approximately 36 mg/g of total phenolics and approximately 19.2 mg/g of anthocyanins.	A total of 37 participants (13 men and 24 women), aged between 60 and 75 years, were evaluated. Inclusion criteria comprised a body mass index (BMI) between 18.5 and 29.9 kg/m^2^, adequate visual acuity, fluency in English, the ability to walk unassisted for 20 min, and, for female participants, a postmenopausal status of at least 12 months.	The study demonstrated that the inclusion of small amounts of blueberries in the diets of older adults may improve specific aspects of cognitive performance. Participants committed fewer errors on switch trials across study visits (F(2,70) = 7.49, *p* = 0.001). Analysis of errors on switch stimuli revealed a significant intervention group × visit interaction (F(2,70) = 3.59, *p* = 0.033, ηp^2^ = 0.09), indicating that participants in the blueberry group exhibited a greater reduction in switch-related errors over time compared with those in the control group.
(Miller, M.G.; Thangthaeng, N.; Rutledge, G.A.; Scott, T.M.; Shukitt-Hale, B., 2021) [91]	Strawberry	24 g/day, equivalent to two cups per serving of fresh Strawberry (SB). 90 days The phenolic composition of SB was not described in the experiment.	A total of 338 participants aged 60–75 years were included. Eligibility criteria comprised a body mass index (BMI) between 18.5 and 29.9 kg/m^2^, the ability to walk unassisted for 20 min, fluency in English, self-reported adequate visual acuity, and, for female participants, a postmenopausal status of more than 12 months.	In this study, participants completed the California Verbal Learning Test at baseline and again after 90 days of dietary intervention with either strawberries (SB) or placebo. At baseline, participants randomized to the SB group recognized fewer words than those assigned to the placebo group. Following the intervention, participants in the SB group demonstrated improved word recognition performance, whereas no change was observed in the placebo group. These findings suggest that the inclusion of strawberries in the diet may help preserve specific aspects of hippocampal-dependent cognitive function during normal aging. Diet may also represent an important modifiable factor in reducing the risk of Alzheimer’s disease and related dementias. With respect to anthropometric outcomes, no significant differences were observed between groups in body weight, waist circumference, or vital signs.
(Nilsson, A.; Salo, I.; Plaza, M.; Björck, I., 2017) [92]	Berry Beverage (Blueberries, blackcurrant, elderberry, lingonberries, strawberry, tomatoes)	Red fruit drink based on a mixture of red fruits (150 g blueberries, 50 g black currants, 50 g elderberries, 50 g bilberries, 50 g strawberries and 100 g tomatoes) daily for 5 weeks. The characterization of red fruit drinks was: Total polyphenols 1324.9 (mg/L), Anthocyanins (mg/L) 414.2, Flavanols (mg/L) 155.9.	A total of 40 apparently healthy adults aged 50–70 years were enrolled. Inclusion criteria comprised non-smoking status, age between 50 and 70 years, and normal to mildly elevated body mass index (BMI ≤ 28 kg/m^2^). Exclusion criteria included fasting blood glucose levels > 6.1 mmol/L, diagnosed metabolic disorders, food allergies, gastrointestinal diseases, or known cognitive disorders that could interfere with study outcomes. Owing to the structure of the cognitive assessments, participants were required to be fluent in Swedish.	Consumption of the red fruit blend for five weeks resulted in reductions in total cholesterol and low-density lipoprotein cholesterol (LDL-C) and prevented monosaccharide-induced impairments in glucose homeostasis and insulin sensitivity. In parallel, an improvement in working memory capacity was observed. The combined improvements in cardiometabolic risk markers and cognitive performance following berry drink consumption support the potential preventive role of berries in relation to type 2 diabetes, cardiovascular disease, and associated cognitive decline.
(Lopresti, A.L.; Smith, S.J.; Pouchieu, C.; PourTAU, L.; Gaudout, D.; Pallet, V.; Drummond, P.D., 2023) [93]	*Vitis vinifera* L. extract	150 mg twice daily (capsules); 300 mg per day. No information on the composition of the extract. This daily dose is equivalent to eating approximately 185 g of grapes (35 to 40 grapes) or 34 g of blueberries (65 to 70 blueberries) per day. The study period was 6 months.	A total of 143 volunteers aged 60–80 years were included. Eligibility criteria comprised male and female participants with self-reported attention and memory difficulties and Montreal Cognitive Assessment–Basic Version (MoCA-BV) scores ranging from 13 to 18. Participants were required to live independently, be non-smokers, have a body mass index (BMI) between 18 and 30 kg/m^2^, and have no plans to initiate new medical treatments during the study period.	Extract supplementation was associated with greater improvements in information processing speed, performance on the Brief Test of Attention (Brief-A), visuospatial learning, and the overall BRIEF-A score. Within the extract group, correlational analyses indicated that changes in dietary polyphenol intake from baseline to week 24 were not significantly associated with changes in episodic memory (r = −0.106, *p* = 0.409), working memory (r = 0.096, *p* = 0.452), information processing speed (r = −0.017, *p* = 0.895), or attentional accuracy (r = −0.124, *p* = 0.334). These findings suggest that background dietary polyphenol intake did not influence the cognitive-enhancing effects of the extract over the intervention period. Anti-obesity outcomes were not assessed in this study.
(Dodd, G.F.; Williams, C.M.; Butler, L.T.; Spencer, J.P., 2019) [94]	Blueberry Beverage	Flavonoid-rich blueberry drink (579 mg anthocyanidins and procyanidins), 30 g per drink, a single visit by the researcher, 2 and 5 h after consumption.	A total of 18 participants (10 women and 8 men) with Mini-Mental State Examination (MMSE) scores ≤ 25 were included. Cognitive function was assessed at baseline and at 2 and 5 h following the intervention.	Cognitive performance differed significantly between 2 and 5 h following consumption of the control beverage (*p* < 0.05), with a decline relative to baseline observed at 2 h. In contrast, cognitive function improved following the blueberry beverage at both post-intervention time points. A trend toward attenuation of the postprandial increase in systolic blood pressure was also observed following the blueberry beverage compared with the control beverage (*p* = 0.08). Plasma brain-derived neurotrophic factor (BDNF) concentrations decreased after consumption of the control beverage; this reduction was attenuated following the blueberry beverage, although the difference did not reach statistical significance (*p* > 0.05). Anti-obesity outcomes were not assessed in this study.
(Whyte, A.R.; Cheng, N.; Butler, L.T.; Lamport, D.J.; Williams, C.M., 2019) [95]	blueberry, strawberry, raspberry, and blackberry	400 mL of ‘smoothie’ containing 75 g of whole strawberries, blueberries, blackberries and raspberries, mixed with 100 mL of water and containing 14.3 g of polyphenols. Important: Average flavonoid content mg/75 g by flavonoid class and berry type. It was 2 days of study and observation.	A total of 46 healthy young adults were recruited for the study. Although specific inclusion criteria were not explicitly reported, the study defined several exclusion criteria, including non-native English speakers, significant visual, auditory, or language impairments, medical conditions such as diabetes or cardiovascular disease, and pregnancy.	Participants in the berry condition demonstrated higher accuracy than those receiving placebo. Post hoc analyses indicated no significant differences between interventions on incongruent trials at 2 and 4 h post-intervention; however, accuracy on incongruent trials was significantly higher in the berry condition at 6 h compared with placebo (*p* = 0.002). In addition, accuracy in the placebo condition on incongruent trials declined significantly between 2 and 6 h and between 4 and 6 h (both *p* < 0.001), whereas performance in the berry condition remained stable throughout the day. These findings suggest that the observed intervention × session interaction effects were primarily driven by deteriorating placebo performance on cognitively demanding incongruent trials. Anti-obesity outcomes were not assessed in this study.
(Curtis, P.J.; van der Velpen, V.; Berends, L.; Jennings, A.; Haag, L.; Minihane, A.M.; Chandra, P.; Kay, C.D.; Rimm, E.B.; Cassidy, A., 2024) [96]	Blueberry powder	1 cup of blueberries, 150 g, presented in powder form; (equivalent to 1 cup of fresh blueberries; 364 mg of anthocyanin and 879 mg of phenolics); Total study time: 6 months.	A total of 138 participants were eligible for inclusion. The study population comprised adults aged 50–75 years who were classified as overweight or obese, with a body mass index (BMI) ≥ 25 kg/m^2^	During the 6-month intervention period, no statistically significant differences were observed between groups across any domain of cognitive function (*p* > 0.05). However, consumption of one cup of blueberries per day was associated with a trend toward improved image recognition accuracy, corresponding to a 4.2% increase (*p* = 0.10; q = 0.59). Similarly, changes in self-reported alertness and mood did not differ significantly between intervention groups following chronic blueberry intake (*p* > 0.05), although alertness approached statistical significance among participants consuming one cup per day (*p* = 0.08; q = 0.24).
(Whyte, A.R.; Rahman, S.; Bell, L.; Edirisinghe, I.; Krikorian, R.; Williams, C.M.; Burton-Freeman, B., 2021) [97]	Wild Blueberry	The wild blueberry drink consisted of 25 g of freeze-dried whole wild blueberry (WBB) powder (~1 cup fresh weight). The bioactive substances contained in this drink were: 725 mg of Polyphenols, 475 mg of Anthocyanins. Time of study: 8 h (after meal).	A total of 35 adults aged 40–65 years participated in this human study. Eligibility criteria included a body mass index (BMI) between 18.5 and 34.9 kg/m^2^, non-smoking status for at least two years, and the ability to understand and complete cognitive function tasks. Participants were required not to be using medications that could interfere with study outcomes, including glucose-lowering, lipid-lowering, or psychostimulant drugs, and to have no history of cardiovascular, respiratory, renal, gastrointestinal, or neurological disorders.	Significant effects on the Auditory Verbal Learning Task (AVLT) were observed exclusively for the recognition memory component, in which participants were required to identify previously presented words from a list of 50 items. A significant main effect of beverage was detected (F(1,39.5) = 6.65, *p* = 0.014), with participants receiving wild blueberry (WBB) demonstrating higher recognition accuracy (M = 0.825) compared with those receiving placebo (M = 0.80). The Beverage × Time interaction showed a trend toward significance (F(4,70) = 2.01, *p* = 0.094), and pairwise comparisons indicated superior performance following WBB relative to placebo, most notably at 240 min post-consumption (*p* = 0.002). This study also assessed body mass index (BMI) as an obesity-related indicator. Metabolic responses differed between treatments during the first 120 min postprandially, with significantly lower glucose and insulin concentrations observed following WBB compared with placebo. When metabolic variables, BMI, and age were included as covariates, their impact varied according to the cognitive outcome examined. BMI emerged as a significant predictor or interaction term for specific measures, including Immediate Recall (trend: F(1,38.4) = 3.89, *p* = 0.056) and Total Number of Words Learned, where a BMI × Beverage interaction was observed (F(1,34.3) = 3.90, *p* = 0.056). In both the WBB and placebo conditions, higher BMI was associated with poorer performance (WBB β = −0.139; placebo β = −0.032).
(Cheatham, C.L.; Canipe, L.G., 3rd; Millsap, G.; Stegall, J.M.; Chai, S.C.; Sheppard, K.W.; Lila, M.A., 2023) [98]	Wild blueberries	35 g of wild blueberry powder per day (equivalent to about one serving of berries); equivalent to about 178 g of fresh blueberries. This dose contained approximately: 302 mg of anthocyanins, responsible for many of the bioactive effects of blueberries; 202 mg of chlorogenic acid, another phenolic compound with antioxidant activity.	A randomized, double-blind, placebo-controlled clinical trial was conducted in older adults aged 65–80 years with mild cognitive impairment, including 44 participants allocated to the blueberry intervention group and 42 to the placebo group, alongside a healthy control group (n = 45). Cognitive outcomes were assessed using the Cambridge Neuropsychological Test Automated Battery (CANTAB) to evaluate information processing performance, particularly processing speed. In addition, event-related potentials (ERP) were recorded as electrophysiological measures of neural processing speed.	Performance on the Rapid Visual Processing task, a component of the Cambridge Neuropsychological Test Automated Battery (CANTAB), improved significantly in participants who consumed wild blueberries for six months. The magnitude of improvement was sufficient for the blueberry group to recover processing speed to levels comparable with those of a healthy reference group, whereas no comparable improvement was observed in the placebo group. Electrophysiological measures further supported these findings, demonstrating enhanced neural processing speed in the blueberry group relative to placebo. Notably, these effects were most pronounced among participants aged 75–80 years, suggesting a greater cognitive benefit in the older subgroup.
(Delaney, K.; Tsang, M.; Kern, M.; Rayo, V.U.; Jason, N.; Hong, M.Y.; Liu, C.; Hooshmand, S., 2025) [99]	Strawberries	Strawberries (in freeze-dried powder form, equivalent to 2 cups of fresh strawberries). Daily dose: 26 g of freeze-dried strawberry powder, equivalent to about 2 cups of fresh strawberries. 26 g of powder contains approximately 73.6 mg of anthocyanins.	A randomized, double-blind, placebo-controlled, crossover clinical trial was conducted in 35 healthy older adults with a mean age of 72 ± 6 years, including 17 women and 18 men. Participants had a mean body mass index (BMI) of 26.4 ± 3.9 kg/m^2^, corresponding to the overweight range. Cognitive function was assessed using validated tests from the NIH Toolbox, including measures of processing speed and episodic memory. In addition, cardiovascular and metabolic parameters were evaluated, including systolic blood pressure (SBP), waist circumference, triglyceride levels, total antioxidant capacity, lipid profile, and insulin concentrations.	Cognitive processing speed improved significantly during the strawberry consumption phase (*p* < 0.001), whereas episodic memory showed greater improvement during the control phase (*p* = 0.002). Systolic blood pressure (SBP) was significantly reduced during strawberry intake (*p* = 0.044). Waist circumference exhibited a significant main effect of reduction over time (*p* = 0.043), although this effect was not phase dependent. Triglyceride levels increased during the control phase (*p* = 0.012) but remained stable during the strawberry phase. Total antioxidant capacity decreased during the control phase (*p* = 0.032) and increased significantly during strawberry consumption (*p* = 0.047). No significant differences were observed between phases or over time for total cholesterol, HDL cholesterol, LDL cholesterol, glucose, diastolic blood pressure, C-reactive protein (CRP), or insulin levels.

A formal assessment of the methodological quality of the included human clinical trials was conducted and is summarized in Table 3. Risk of bias was evaluated using the Cochrane Risk of Bias 2 (RoB 2) tool, which is recommended for the assessment of randomized clinical trials. This evaluation focused on identifying the main sources of potential bias across studies, including small sample sizes, short intervention durations, participant attrition, selective outcome reporting, and the use of multiple cognitive assessments without appropriate adjustment. The results of this assessment were considered in the interpretation of the overall findings. Regarding the overall risk of bias, 12 studies were considered to have “some concerns” and only one was considered to have “low risk”.

The methodological quality of the observational study was assessed using the Newcastle–Ottawa Scale (NOS) for cohort studies. This tool evaluates three domains: selection of the cohort, comparability of study groups, and assurance of outcomes. Studies can receive a maximum of nine stars, with higher scores indicating lower risk of bias (Table 4).

## 4. Discussion

This review integrates more than a decade of research examining the effects of berry consumption on cognitive function and obesity—two closely interconnected domains that have driven increasing scientific interest. The included studies collectively explore mechanisms through which excess body weight and cognitive decline may be modulated, including gut microbiome dysbiosis, alterations in blood–brain barrier permeability, increased release of pro-inflammatory cytokines, and changes in the expression and distribution of adipokines. Together, these pathways provide a mechanistic framework linking metabolic dysfunction to cognitive impairment and support the investigation of berry-derived bioactive compounds as potential modulators of these processes.

In this systematic review, the results were derived exclusively from human clinical studies; however, as outlined in the Methods Section (Section 2), evidence from laboratory animal studies was selectively referenced to support mechanistic discussions related to berry consumption, obesity, and cognitive outcomes.

Polyphenols present in berries have garnered considerable interest due to their dual capacity to influence both neural and metabolic health. These compounds—particularly anthocyanins and other flavonoids—exert antioxidant and anti-inflammatory effects that may protect neuronal integrity and support synaptic plasticity. Concurrently, they modulate glucose homeostasis and lipid metabolism, contributing to body weight regulation. This convergence of neuroprotective and metabolic actions positions berries as promising candidates for dietary strategies aimed at addressing obesity and cognitive decline simultaneously. Human clinical studies are especially critical for validating mechanisms identified in preclinical models and for determining effective dosages, intervention durations, and long-term safety profiles. Ultimately, rigorous clinical evaluation of berry-derived polyphenols may inform evidence-based public health strategies, with potential implications for reducing the burden of dementia and obesity in aging populations.

### 4.1. MCI and Biomarkers

Several of the studies included in this review reported improvements in cognitive performance, with memory outcomes being the most frequently assessed and the most consistently affected domain. Across studies, inclusion criteria commonly incorporated body mass index (BMI), older age (generally ≥60 years), and baseline cognitive status, typically characterized using standardized measures such as mild cognitive impairment (MCI) criteria, memory tests, and verbal fluency assessments. Importantly, baseline cognitive impairment was generally mild, allowing for the detection of intervention-related effects. However, the near-exclusive focus on memory highlights a notable gap in the literature, as other cognitive domains—such as attention, executive function, and processing speed—remain underexplored. In addition to cognitive outcomes, several studies assessed biological markers, including lipid profiles, brain-derived neurotrophic factor (BDNF), neurofilament light chain, TAU protein, and amyloid-beta. Notably, only one study evaluated inflammatory proteins, despite the well-established relevance of inflammatory pathways in the interplay between obesity and mild cognitive impairment. This limitation underscores the need for more comprehensive biomarker profiling in future clinical trials.

With respect to mild cognitive impairment (MCI), a recognized prodromal stage of dementia, the potential protective role of anthocyanins against Alzheimer’s disease (AD) has been highlighted by their capacity to modulate pathways involved in disease progression. Consistent with this notion, epidemiological and clinical evidence indicates that regular consumption of berries, vegetables, and polyphenol-rich beverages is associated with a reduced risk of age-related neurological disorders, including AD [100]. Importantly, the neuroprotective effects of berries appear to be more evident during pre-dementia stages, when there is a gradual accumulation of senile plaques and neurofibrillary tangles. At this stage, bioactive compounds may exert effects that limit amyloid-beta accumulation and attenuate TAU protein hyperphosphorylation. However, it should be noted that a portion of the supporting evidence derives from preclinical models and from polyphenols not exclusively sourced from red fruits, underscoring the need for further well-designed human studies to clarify the specificity and translational relevance of these findings.

### 4.2. Berries, Gut Microbiota and MCI/Alzheimer Biomarkers

Recent preclinical studies in murine models suggest that anthocyanins exert beneficial effects on gut microbiota composition and function by promoting the growth of beneficial bacterial taxa and inhibiting the proliferation of potentially pathogenic species. This modulation of the gut microbiome may, in turn, influence neurotransmitter synthesis and signaling along the gut–brain axis, with potential implications for cognitive function and behavior [101]. Evidence further indicates that the gut microbiota plays a regulatory role in the hypothalamic–pituitary–adrenal (HPA) axis, a central mediator of the stress response. Alterations in microbial composition have been shown to affect HPA axis reactivity, which may contribute to stress-related cognitive impairments. In addition, the gut microbiome is capable of producing neuroactive compounds, including serotonin, dopamine, and gamma-aminobutyric acid (GABA), all of which are critical for normal brain function and mood regulation.

A recent investigation examined the relationship between gut microbiome composition and early cognitive decline. The study analyzed stool samples from 119 individuals with mild cognitive impairment (MCI) and 320 cognitively healthy adults using shotgun metagenomic sequencing to achieve high-resolution microbial profiling. Microbiome data were integrated with established Alzheimer’s disease–related biomarkers, including cerebral amyloid burden assessed by positron emission tomography (PET), plasma levels of phosphorylated TAU (pTAU181), and apolipoprotein E (APOE) genotype. Several microbial taxa exhibited significant associations with these biomarkers, with *Akkermansia muciniphila* notably linked to lower cerebral amyloid load. Functional pathway analyses further suggested that microbial pathways involved in energy metabolism and immune signaling may modulate neurodegenerative processes. Collectively, these findings support a role for specific gut microbial signatures in Alzheimer’s disease pathophysiology and highlight the gut microbiome as a potential target for dietary or therapeutic strategies aimed at delaying cognitive decline [102].

Gut bacteria produce a range of metabolites, including short-chain fatty acids (SCFAs), which can influence immune function, central nervous system activity, and, in some cases, cross the blood–brain barrier, thereby potentially affecting cognitive processes [103]. Dietary patterns rich in polyphenols, such as those derived from berries, may modulate the production and biological effects of these microbial metabolites by shaping gut microbiota composition and function. Across the studies included in this review, clinical trials administering bioactive compounds at higher or more standardized concentrations tended to report more pronounced biological and cognitive effects, underscoring the importance of dose, formulation, and bioavailability in evaluating the efficacy of berry-derived interventions.

Bioactive flavonoid pigments found in red and purple fruits have been shown to inhibit beta-amyloid (Aβ) peptide aggregation and tau protein hyperphosphorylation, two central hallmarks of Alzheimer’s disease (AD) pathology that are increasingly associated with alterations along the gut–brain axis [104]. In line with this evidence, recent studies in rodent models have demonstrated that these compounds exhibit anti-amyloidogenic and anti-tau properties, supporting their potential therapeutic relevance in mitigating AD-related pathological features. Specifically, they have been shown to inhibit the aggregation of Aβ peptides into oligomeric and fibrillar species, thereby reducing amyloid plaque formation in the brain [105]. Advances in the understanding of AD pathophysiology have enabled the identification of key biomarkers associated with senile plaque deposition and neurofibrillary tangle formation—namely Aβ peptides and tau protein. Within this framework, anthocyanins appear to exert biological activity against these molecular targets, as well as against additional biomarkers implicated in dementia progression.

Anthocyanins (ACNs) have been shown to attenuate TAU protein hyperphosphorylation, a critical event in the development of neurofibrillary pathology, by modulating the activity of key kinases involved in TAU phosphorylation. In addition, ACNs and ACN-rich plant extracts have been reported to inhibit amyloid-beta (Aβ) accumulation and to exert neuroprotective effects against neurodegeneration [106]. As discussed in the literature, kinases—enzymes that regulate cellular processes through phosphorylation—play a central role in the onset and progression of Alzheimer’s disease (AD). Dysregulation of several kinase pathways contributes not only to the abnormal accumulation of Aβ and TAU proteins but also to neuroinflammatory responses and synaptic dysfunction, further exacerbating cognitive decline.

Evidence from in vitro studies indicates that insulin resistance plays a critical role in TAU protein hyperphosphorylation. Under physiological conditions, intact insulin signaling promotes phosphorylation of the insulin receptor substrate (IRS) at tyrosine residues, leading to activation of protein kinase B (AKT), which subsequently inhibits glycogen synthase kinase-3 (GSK-3), thereby preventing TAU phosphorylation [76,78]. Based on the studies evaluated in this review, insulin resistance emerges as an important contributing factor to TAU hyperphosphorylation. Disruption of insulin signaling in the brain alters the balance between TAU kinases and phosphatases, resulting in increased kinase activity and reduced phosphatase function. This imbalance promotes TAU detachment from microtubules and facilitates the formation of neurofibrillary tangles, a hallmark of neurodegenerative pathology.

Foods and their associated bioactive compounds have been increasingly recognized as modulators of chronic disease processes, in part due to their generally favorable safety profiles when compared with pharmacological therapies, which may be associated with adverse effects, as demonstrated in preclinical models. The beneficial effects of dietary anthocyanins have been described in the context of metabolic disorders and obesity-related inflammation, with evidence of modulation of metabolic and inflammatory markers in both animal models and human cellular systems [107]. Importantly, metabolic dysfunction and obesity-induced inflammation have been proposed as early events preceding the clinical manifestation of mild cognitive impairment (MCI), potentially presenting initially as subjective cognitive complaints. Within this framework, the identification of dietary agents at biologically effective concentrations capable of mitigating these early metabolic and inflammatory alterations represents a topic of significant scientific and clinical interest.

**Figure 5:**
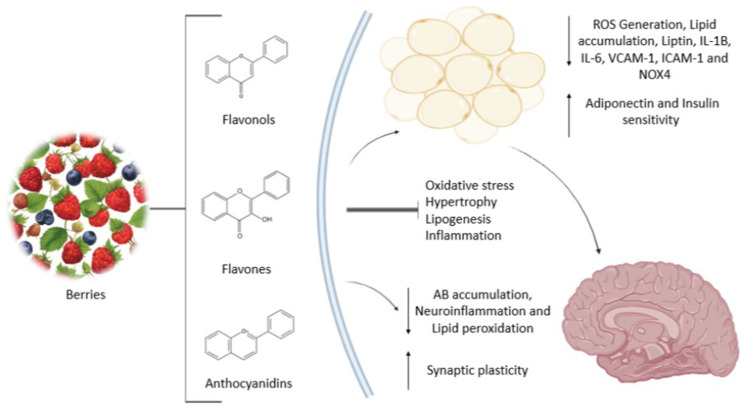
Relationship between fruit composition and its effects, with the action of bioactive compounds on pathways related to obesity and cognitive decline. AB: beta amyloid; ROS: reactive oxygen species; IL-1B: interleukin 1-beta; IL-6: interleukin-6; VCAM-1: vascular cell adhesion Molecule-1; ICAM-1: intercellular adhesion molecule-1; NOX4: NADPH oxidase 4.

Figure 5 schematic representation of the proposed effects of red berry consumption on obesity-related pathways, including modulation of inflammation, oxidative stress, and free radical generation. These biological actions may contribute to the attenuation of mild cognitive impairment (MCI), a recognized pre-dementia stage, thereby supporting the potential role of red berries as a dietary strategy for reducing dementia risk [108].

### 4.3. The Role of Adipocytes

Adipokines are bioactive molecules produced and secreted by adipocytes that play key roles in regulating inflammatory and metabolic processes. Among the most extensively studied adipokines are tumor necrosis factor-alpha (TNF-α), leptin, resistin, visfatin, interleukin-6 (IL-6), and adiponectin [109]. A distinguishing feature among the more than 50 currently identified adipokines is their differential involvement in pro- or anti-inflammatory signaling. Alterations in adipokine secretion profiles have been consistently observed across body mass index (BMI) categories: individuals with obesity tend to exhibit adipose tissue characterized by predominant secretion of pro-inflammatory adipokines, whereas lean individuals more frequently display a profile enriched in anti-inflammatory adipokines [110]. Notably, none of the clinical studies included in this review directly assessed adipokine concentrations, although some evaluated circulating pro-inflammatory proteins. Among adipokines, leptin warrants particular attention, as it is centrally involved in appetite regulation and energy balance. Elevated leptin levels commonly observed in obesity may reflect leptin resistance, a condition that has been associated with impaired central nervous system signaling and potential adverse effects on cognitive function.

Although this review primarily focuses on berries, consideration of grape-derived extracts and other polyphenol-rich foods as relevant analogues is justified. These sources provide comparable profiles of bioactive polyphenols—particularly anthocyanins and flavonoids—that act through shared biological pathways involving oxidative stress modulation, inflammatory regulation, and metabolic control. From a mechanistic standpoint, inclusion of these analogues supports a broader interpretation of how fruit-derived polyphenols may influence obesity-related dysfunction and cognitive processes. This perspective enhances conceptual coherence and translational relevance while maintaining the central emphasis on berries.

A preregistered systematic review and meta-analysis including 42 cross-sectional and 13 longitudinal studies investigated the associations between circulating adipokines—leptin, adiponectin, resistin, and ghrelin—and the prevalence of all-cause dementia, Alzheimer’s disease (AD), and mild cognitive impairment (MCI) [111]. The analysis demonstrated that individuals with AD exhibited lower circulating leptin levels and higher resistin levels compared with cognitively normal participants. Moreover, reduced leptin concentrations and elevated resistin levels were associated with greater severity of cognitive impairment. Importantly, lower leptin levels in later life were linked to an increased prospective risk of dementia and AD. In contrast, findings related to ghrelin and adiponectin were inconclusive, with age, sex distribution, obesity status, and dementia severity acting as moderating factors in several analyses. Collectively, these findings underscore that biomarkers relevant to MCI and dementia are not limited to classical neuropathological markers such as TAU or amyloid-beta. Adipocyte-derived biomarkers may provide valuable complementary diagnostic and prognostic information, reinforcing the close interrelationship between obesity, inflammation, and cognitive impairment.

**Figure 6:**
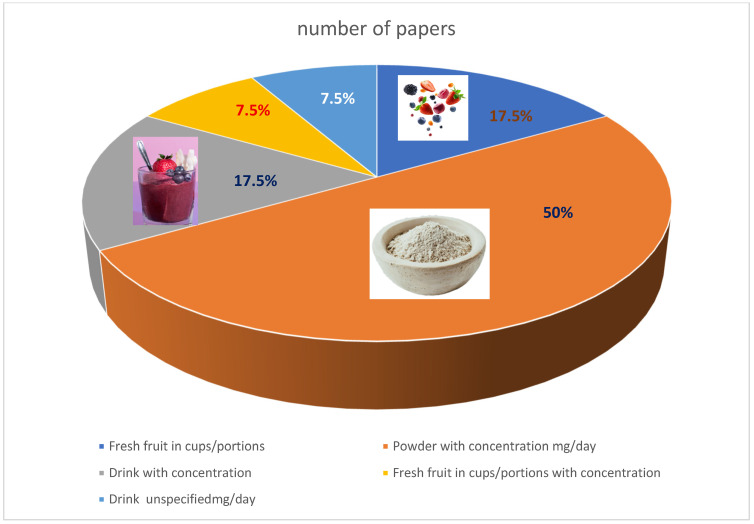
Available bioactives.

Fruits can be consumed in multiple forms and processed using diverse methods, including fresh portions, juices, smoothies, frozen products, and freeze-dried fruit powders [112]. Across the studies included in this review, substantial variability was observed in the formulation and delivery of bioactive compounds, ranging from whole fresh fruit provided in standardized servings to freeze-dried extracts administered in powder form, with detailed reporting of compound concentrations (Figure 6). This heterogeneity underscores the importance of clearly identifying which bioactive constituents are being evaluated, as well as their respective doses and formulations, to enable accurate interpretation and comparison of study outcomes. Standardization of bioactive characterization and dosing is therefore critical for strengthening the consistency and reproducibility of findings in this field.

Clinical trials published in 2024 demonstrated that acute intake of wild blueberry extract—particularly at a dose of 222 mg of anthocyanins—can attenuate the postprandial decline in executive function commonly observed in older adults. Participants receiving the extract exhibited faster reaction times and greater cognitive stability compared with placebo, indicating immediate, short-term cognitive benefits. Although these effects were acute rather than sustained, the findings suggest a potential role for blueberry-derived bioactives in supporting day-to-day cognitive performance [85]. In parallel, a 2025 pilot study evaluating six months of elderberry juice supplementation in individuals with mild cognitive impairment reported encouraging cognitive outcomes. Participants in the intervention group demonstrated shorter response times on tasks assessing cognitive flexibility, consistent with improved executive processing. While these results were preliminary and derived from a limited sample size, they support a possible neuroprotective role for anthocyanin-rich elderberry. Collectively, these findings strengthen the rationale for dietary interventions targeting cognitive decline through polyphenol-rich berries. Nonetheless, larger, well-powered clinical trials with longer follow-up are required to confirm efficacy and establish long-term benefits [113].

Regarding the bioactive compounds summarized in Table 2—particularly anthocyanins, flavonoids, and related polyphenols—it is evident that berries may exert distinct biological effects depending on the concentration and profile of these constituents. For example, strawberry consumption has been associated with improvements in cognitive performance and cardiovascular health, likely reflecting its nutrient composition and antioxidant capacity [59]. Similarly, blueberry intake has been consistently linked to reductions in oxidative stress and inhibition of inflammatory processes [114]. A randomized crossover trial evaluating the effects of jaboticaba peel powder supplementation (7 g/day) in 19 healthy adults demonstrated significant metabolic and cognitive benefits. The intervention delivered high levels of polyphenols, including cyanidin-3-O-glucoside and ellagic acid, and resulted in significant reductions in interleukin-6 (IL-6) levels and postprandial reactive oxygen species, indicating anti-inflammatory and antioxidant activity. In addition, participants exhibited improved postprandial selective attention compared with the control condition, suggesting cognitive benefits following four weeks of supplementation [115]. Other berries—including haskap, black raspberry, blackcurrant, elderberry, and lingonberry—are similarly rich in anthocyanins and polyphenols and have demonstrated neuroprotective potential. These compounds have been shown to reduce oxidative stress, attenuate neuroinflammation, and enhance synaptic plasticity through modulation of signaling pathways such as brain-derived neurotrophic factor (BDNF) and cAMP response element-binding protein (CREB). Evidence from both human and preclinical studies further indicates that bioactive metabolites derived from these berries can cross the blood–brain barrier, supporting neuronal survival, mitochondrial function, and vascular health. Collectively, these findings position anthocyanin-rich berries as promising dietary components for delaying cognitive decline and promoting healthy brain aging [116].

### 4.4. The Risk of Bias

The overall risk of bias across the included randomized controlled trials was predominantly rated as “some concerns” according to the Cochrane Risk of Bias 2 (RoB 2) tool. This classification was primarily driven by incomplete reporting of randomization procedures, absence of prospective protocol registration in some trials, and limited information regarding allocation concealment—methodological challenges that are frequently encountered in nutritional intervention research. Importantly, none of the included studies was judged to be at high risk of bias in any domain, and one trial was rated as low risk across all assessed domains, thereby strengthening overall confidence in the evidence base. Most trials adequately controlled for deviations from intended interventions and employed validated, objective cognitive outcome measures, resulting in a low risk of bias related to outcome measurement. Missing outcome data constituted a source of some concern in several studies, largely due to participant attrition inherent to dietary interventions and crossover designs; however, attrition rates were generally balanced between intervention groups and were unlikely to have materially influenced the direction of the reported effects. Taken together, although the presence of methodological concerns warrants cautious interpretation, the consistency of findings across studies with heterogeneous designs, populations, and intervention durations suggests that the observed cognitive benefits associated with berry-based interventions are unlikely to be attributable solely to bias. Future trials would benefit from greater transparency in randomization and allocation procedures, prospective trial registration, and standardized reporting of primary outcomes to further strengthen the quality and reproducibility of evidence in this field.

The observational evidence included in this review was judged to be of high methodological quality based on assessment with the Newcastle–Ottawa Scale (NOS). Notably, the prospective cohort study conducted by Devore et al. achieved the maximum NOS score, reflecting strong cohort representativeness, rigorous dietary exposure assessment using repeated validated food frequency questionnaires, comprehensive control of relevant confounders, and robust ascertainment of cognitive outcomes over long-term follow-up. Although residual confounding cannot be entirely excluded due to the observational nature of the study, the consistency of the observed associations following extensive multivariable adjustment, together with the large sample size, supports the internal validity of the findings. Importantly, the high methodological quality of this cohort study complements the evidence derived from randomized controlled trials, thereby strengthening the overall inference regarding the association between berry-derived flavonoid intake and cognitive aging.

This systematic review focused on evaluating the influence of polyphenol intake derived from berry consumption on obesity-related mechanisms and their association with cognitive performance. The included clinical trials reported measurable changes in cognitive and metabolic outcomes, providing the empirical basis for the interpretations discussed in this manuscript. Collectively, these findings support the relevance of berry-derived polyphenols as dietary components capable of modulating metabolic health and cognitive function, particularly in populations at increased risk of obesity-associated cognitive decline.

The integration of both cognitive and metabolic outcomes in this review adds value relative to previous syntheses on berries and cognition, as it focuses exclusively on human studies and examines associations across cognitive domains in relation to dose, bioactive compound concentration, berry type, and multiple metabolic and cognitive parameters. Notably, several studies summarized in Table 2 illustrate these multidimensional relationships. For example, the study by Whyte et al. (2021) [97] which investigated wild blueberry supplementation, reported favorable metabolic changes, including reductions in body mass index and significantly lower glucose and insulin levels. Importantly, these metabolic improvements were accompanied by significant cognitive effects on the Auditory Verbal Learning Task (AVLT), specifically within the recognition memory component. This finding underscores the potential interdependence between metabolic regulation and selective cognitive domains, supporting a more integrative interpretation of berry-derived polyphenol effects on cognitive aging.

The clinical trials included in this review were conducted exclusively in human participants and aimed to evaluate evidence regarding the effects of bioactive compounds on cognitive function, irrespective of a formal pre-dementia diagnosis. In this context, the presence or absence of mild cognitive impairment (MCI) was not treated as a mandatory inclusion criterion. The investigated bioactive compounds were derived from a limited range of sources, primarily blueberries, strawberries, raspberries, grapes, and other berries. Across the literature, a recurring research objective has been to elucidate the relationship between obesity-related mechanisms, cognitive decline—including MCI—and the neuroprotective potential of berry-derived bioactive compounds. Collectively, these studies reflect a growing interest in understanding how dietary polyphenols may modulate metabolic dysfunction and cognitive outcomes within a unified biological framework.

## 5. Conclusions

Results derived from the analysis of human clinical trials suggest that phenolic compounds present in red fruits may exert beneficial effects in specific cognitive domains, particularly memory, language, and executive function. In parallel, these compounds have demonstrated potential to favorably influence energy metabolism, with preliminary evidence of a possible role in modulating pathways related to obesity, including the regulation of adipokines. However, current evidence remains limited and inconclusive. Important uncertainties persist regarding the magnitude, consistency, and clinical relevance of these effects. In particular, the ideal concentrations of phenolic compounds capable of attenuating tau protein hyperphosphorylation, reducing beta-amyloid accumulation, modulating leptin signaling, and improving insulin sensitivity have not yet been clearly established. The most robust evidence for the reduction in tau protein hyperphosphorylation and Aβ accumulation comes from preclinical models and, in some cases, from polyphenols not restricted to red fruits, while the human clinical trials summarized here have not yet demonstrated significant effects on these biomarkers. Filling these gaps represents a fundamental objective for future large-scale, well-controlled clinical trials in humans, which will be essential to substantiate the mechanistic relationships and define the translational potential of phenolic compounds derived from red fruits in preventing cognitive decline associated with obesity.

## Figures and Tables

**Table 1:** PICOS criteria for inclusion of studies.

Population	Young People and Adults with No Age Restrictions
Intervention	Berries containing anthocyanins are offered as part of the diet or as a supplement, for a period of more than 2 h up to a maximum of 3 years
Comparator	The exposure was based on habitual intake/quantiles and quartiles of wild berry consumption and/or in the form of extracts
Outcomes	In isolation or in combination: cognition, memory, language, verbal flow; Body mass index
Study types	randomized double blind; Prospective cohort study

**Table 3:** Risk of bias assessment of included randomized controlled trials using the Cochrane RoB 2 tool.

Study	Bias Arising from the Randomization Process	Bias Due to Deviations from Intended Interventions	Bias Due to Missing Outcome Data	Bias in Measurement of the Outcome	Bias in Selection of the Reported Result	Overall Risk of Bias
Delaney, K.; Tsang, M.; Kern, M.; Rayo, V.U.; Jason, N.; Hong, M.Y.; Liu, C.; Hooshmand, S., 2025 [99]	Low risk	Low risk	Some concerns	Low risk	Low risk	Some concerns
Whyte, A.R.; Cheng, N.; Butler, L.T.; Lamport, D.J.; Williams, C.M., 2019 [95]	Some concerns	Some concerns	Low risk	Low risk	Some concerns	Some concerns
Krikorian, R.; Skelton, M.R.; Summer, S.S.; Shidler, M.D.; Sullivan, P.G., 2022 [86]	Low risk	Low risk	Low risk	Low risk	Low risk	Low risk
Wood, E.; Hein, S.; Mesnage, R.; Fernandes, F.; Abhayaratne, N.; Xu, Y.; Zhang, Z.; Bell, L.; Williams, C.; Rodriguez-Mateos, A., 2023 [89]	Some concerns	Low risk	Low risk	Low risk	Low risk	Some concerns
Curtis, P.J.; van der Velpen, V.; Berends, L.; Jennings, A.; Haag, L.; Minihane, A.M.; Chandra, P.; Kay, C.D.; Rimm, E.B.; Cassidy, A., 2024 [96]	Some concerns	Low risk	Some concerns	Low risk	Some concerns	Some concerns
Miller, M.G.; Hamilton, D.A.; Joseph, J.A.; Shukitt-Hale, B., 2018 [90]	Some concerns	Low risk	Some concerns	Low risk	Some concerns	Some concerns
Miller, M.G.; Thangthaeng, N.; Rutledge, G.A.; Scott, T.M.; Shukitt-Hale, B., 2021 [91]	Some concerns	Low risk	Some concerns	Low risk	Some concerns	Some concerns
Nilsson, A.;Salo, I.; Plaza, M.; Björck, I., 2017 [92]	Some concerns	Some concerns	Some concerns	Low risk	Some concerns	Some concerns
Whyte, A.R.; Rahman, S.; Bell, L.; Edirisinghe, I.; Krikorian, R.; Williams, C.M.; Burton-Freeman, B., 2021 [97]	Some concerns	Some concerns	Some concerns	Low risk	Some concerns	Some concerns
Dodd, G.F.; Williams, C.M.; Butler, L.T.; Spencer, J.P., 2019 [94]	Some concerns	Some concerns	Some concerns	Low risk	Some concerns	Some concerns
Lopresti, A.L.; Smith, S.J.; Pouchieu, C.; PourTAU, L.; Gaudout, D.; Pallet, V.; Drummond, P.D., 2023 [93]	Some concerns	Low risk	Some concerns	Low risk	Some concerns	Some concerns
Cheatham, C.L.; Canipe, L.G., 3rd; Millsap, G.; Stegall, J.M.; Chai, S.C.; Sheppard, K.W.; Lila, M.A., 2023) [98]	Some concerns	Low risk	Some concerns	Low risk	Some concerns	Some concerns
Delaney, K.; Tsang, M.; Kern, M.; Rayo, V.U.; Jason, N.; Hong, M.Y.; Liu, C.; Hooshmand, S., 2025 [99]	Some concerns	Some concerns	Some concerns	Some concerns	Some concerns	Some concerns

**Table 4:** Methodological quality assessment of the observational study using the Newcastle–Ottawa Scale (NOS).

Study	Selection (Max 4 Stars)	Comparability (Max 2 Stars)	Outcome (Max 3 Stars)	Total Score	Quality
Devore, E.E.; Kang, J.H.; Breteler, M.M.; Grodstein, F., 2012) [87]	★★★★	★★	★★★	9/9	High quality

## Data Availability

No new data were created or analyzed in this study.

## Supplementary Materials

The following supporting information can be downloaded at: https://www.mdpi.com/article/10.3390/nu18040674/s1, Table S1: PRISMA 2020 checklist.

## References

[1] Petersen R.C. (2016). Mild cognitive impairment. Contin. Lifelong Learn. Neurol..

[2] Eshkoor S.A., Hamid T.A., Mun C.Y., Ng C.K. (2015). Mild cognitive impairment and its management in older people. Clin. Interv. Aging.

[3] Jongsiriyanyong S., Limpawattana P. (2018). Mild Cognitive Impairment in Clinical Practice: A Review Article. Am. J. Alzheimer’s Dis. Other Demen..

[4] Petersen R.C., Lopez O., Armstrong M.J., Getchius T.S.D., Ganguli M., Gloss D., Gronseth G.S., Marson D., Pringsheim T., Day G.S. (2018). Practice guideline update summary: Mild cognitive impairment: Report of the Guideline Development, Dissemination, and Implementation Subcommittee of the American Academy of Neurology. Neurology.

[5] Harada C.N., Natelson Love M.C., Triebel K.L. (2013). Normal cognitive aging. Clin. Geriatr. Med..

[6] Edmonds E.C., Thomas K.R., Rapcsak S.Z., Lindemer S.L., Delano-Wood L., Salmon D.P., Bondi M.W. (2024). Data-driven classification of cognitively normal and mild cognitive impairment subtypes predicts progression in the NACC dataset. Alzheimer’s Dement..

[7] Manly J.J., Jones R.N., Langa K.M., Ryan L.H., Levine D.A., McCammon R., Heeringa S.G., Weir D. (2022). Estimating the prevalence of dementia and mild cognitive impairment in the US: The 2016 Health and Retirement Study Harmonized Cognitive Assessment Protocol Project. JAMA Neurol..

[8] Bradfield N.I. (2023). Mild cognitive impairment: Diagnosis and subtypes. Clin. EEG Neurosci..

[9] Hessen E. (2025). Mild cognitive impairment and neuropsychological examination. Front. Psychol..

[10] Forlenza O.V., Diniz B.S., Gattaz W.F. (2010). Diagnosis and biomarkers of predementia in Alzheimer’s disease. BMC Med..

[11] Shan G., Zhang Y. (2025). Disease progression from mild cognitive impairment to dementia for patients with Alzheimer’s disease or Lewy body pathology. J. Alzheimer’s Dis. Rep..

[12] Herukka S.K., Helisalmi S., Hallikainen M., Tervo S., Soininen H., Pirttilä T. (2007). CSF Abeta42, Tau and phosphorylated Tau, APOE epsilon4 allele and MCI type in progressive MCI. Neurobiol. Aging.

[13] Alzheimer’s Association (2024). Mild Cognitive Impairment (MCI).

[14] Kim B.S., Jun S., Kim H. (2023). Cognitive Trajectories and Associated Biomarkers in Patients with Mild Cognitive Impairment. J. Alzheimer’s Dis..

[15] Mayo Clinic (2024). Mild Cognitive Impairment.

[16] Hugo J., Ganguli M. (2014). Dementia and cognitive impairment: Epidemiology, diagnosis, and treatment. Clin. Geriatr. Med..

[17] Langa K.M., Levine D.A. (2014). The diagnosis and management of mild cognitive impairment: A clinical review. JAMA.

[18] World Obesity Federation (2024). World Obesity Atlas 2024.

[19] Rubino F., Cummings D.E., Eckel R.H., Cohen R.V., Wilding J.P.H., Brown W.A., Stanford F.C., Batterham R.L., Farooqi I.S., Farpour-Lambert N.J. (2025). Definition and diagnostic criteria of clinical obesity. Lancet Diabetes Endocrinol..

[20] Yuan Y., Li J., Zhang N., Fu P., Jing Z., Yu C., Zhao D., Hao W., Zhou C. (2021). Body mass index and mild cognitive impairment among rural older adults in China: The moderating roles of gender and age. BMC Psychiatry.

[21] Quaye E., Galecki A.T., Tilton N., Whitney R., Briceño E.M., Elkind M.S.V., Fitzpatrick A.L., Gottesman R.F., Griswold M., Gross A.L. (2023). Association of Obesity with Cognitive Decline in Black and White Americans. Neurology.

[22] Spencer S.J., Korosi A., Layé S., Shukitt-Hale B., Barrientos R.M. (2017). Food for thought: How nutrition impacts cognition and emotion. npj Sci. Food.

[23] Dye L., Boyle N.B., Champ C., Lawton C. (2017). The relationship between obesity and cognitive health and decline. Proc. Nutr. Soc..

[24] de Bem A.F., Krolow R., Farias H.R., de Rezende V.L., Gelain D.P., Moreira J.C.F., Duarte J.M.D.N., de Oliveira J. (2021). Animal Models of Metabolic Disorders in the Study of Neurodegenerative Diseases: An Overview. Front. Neurosci..

[25] Hata M., Andriessen E.M., Hata M., Diaz-Marin R., Fournier F., Crespo-Garcia S., Sapieha P. (2023). Past history of obesity triggers persistent epigenetic changes in innate immunity and exacerbates neuroinflammation. Science.

[26] Rull A., Camps J., Alonso-Villaverde C., Joven J. (2010). Insulin resistance, inflammation, and obesity: Role of monocyte chemoattractant protein-1 (CCL2) in the regulation of metabolism. Mediat. Inflamm..

[27] Gariballa S., Alkaabi J., Yasin J., Al Essa A. (2019). Total adiponectin in overweight and obese subjects and its response to visceral fat loss. BMC Endocr. Disord..

[28] Kim A.Y., Park Y.J., Pan X., Shin K.C., Kwak S.H., Bassas A.F., Sallam R.M., Park K.S., Alfadda A.A., Xu A. (2015). Obesity-induced DNA hypermethylation of the adiponectin gene mediates insulin resistance. Nat. Commun..

[29] Kong Y., Wang N., Tong Z., Wang D., Wang P., Yang Q., Yan X., Song W., Jin Z., Zhang M. (2024). Role of complement factor D in cardiovascular and metabolic diseases. Front. Immunol..

[30] Grabska-Kobyłecka I., Szpakowski P., Król A., Książek-Winiarek D., Kobyłecki A., Głąbiński A., Nowak D. (2023). Polyphenols and Their Impact on the Prevention of Neurodegenerative Diseases and Development. Nutrients.

[31] Davanzo G.G., Castro G., Monteiro L.B., Castelucci B.G., Jaccomo V.H., da Silva F.C., Marques A.M., Francelin C., de Campos B.B., de Aguiar C.F. (2023). Obesity increases blood-brain barrier permeability and aggravates the mouse model of multiple sclerosis. Mult. Scler. Relat. Disord..

[32] Sonelli F., Fiore M., Chaldakov G., Aloe L. (2007). Adipose tissue-derived nerve growth factor and brain-derived neurotrophic factor: Results from experimental stress and diabetes. Gen. Physiol. Biophys..

[33] Blancas-Flores G., Almanza-Pérez J.C., López-Roa R.I., Alarcón-Aguilar F.J., García-Macedo R., Cruz M. (2010). Obesity as an inflammatory process. Bol. Med. Hosp. Infant. Mex..

[34] Pereira S.S., Alvarez-Leite J.I. (2014). Low-Grade Inflammation, Obesity, and Diabetes. Curr. Obes. Rep..

[35] Chen L., Chen R., Wang H., Liang F. (2015). Mechanisms Linking Inflammation to Insulin Resistance. Int. J. Endocrinol..

[36] Tomkins O., Friedman O., Ivens S., Reiffurth C., Major S., Dreier J.P., Heinemann U., Friedman A. (2007). Blood-brain barrier disruption results in delayed functional and structural alterations in the rat neocortex. Neurobiol. Dis..

[37] Wondmkun Y.T. (2020). Obesity, Insulin Resistance, and Type 2 Diabetes: Associations and Therapeutic Implications. Diabetes Metab. Syndr. Obes..

[38] Bays H.E., Bindlish S., Clayton T.L. (2023). Obesity, diabetes mellitus, and cardiometabolic risk: An Obesity Medicine Association (OMA) Clinical Practice Statement (CPS) 2023. Obes. Pillars.

[39] Brown G.C., Vilalta A. (2015). How microglia kill neurons. Brain Res..

[40] Ngamsamer C., Sirivarasai J., Sutjarit N. (2022). The Benefits of Anthocyanins against Obesity-Induced Inflammation. Biomolecules.

[41] Carabotti M., Scirocco A., Maselli M.A., Severi C. (2015). The gut–brain axis: Interactions between enteric microbiota, central and enteric nervous systems. Ann. Gastroenterol..

[42] Liu Z., Patil I.Y., Jiang T., Sancheti H., Walsh J.P., Stiles B.L., Yin F., Cadenas E. (2015). High-fat diet induces hepatic insulin resistance and impairment of synaptic plasticity. PLoS ONE.

[43] Rogers G.B., Keating D.J., Young R.L., Wong M.L., Licinio J., Wesselingh S. (2016). From gut dysbiosis to altered brain function and mental illness: Mechanisms and pathways. Mol. Psychiatry.

[44] Rusch J.A., Layden B.T., Dugas L.R. (2023). Signalling cognition: The gut microbiota and hypothalamic-pituitary-adrenal axis. Front. Endocrinol..

[45] Aburto M.R., Cryan J.F. (2024). Gastrointestinal and brain barriers: Unlocking gates of communication across the microbiota–gut–brain axis. Nat. Rev. Gastroenterol. Hepatol..

[46] Chen Y., Xu J., Chen Y. (2021). Regulation of neurotransmitters by the gut microbiota and effects on cognition in neurological disorders. Nutrients.

[47] Doifode T., Giridharan V.V., Generoso J.S., Bhatti G., Collodel A., Schulz P.E., Barichello T. (2021). The impact of the microbiota–gut–brain axis on Alzheimer’s disease pathophysiology. Pharmacol. Res..

[48] Bayazid A.B., Kim J.G., Azam S., Jeong S.A., Kim D.H., Park C.W., Lim B.O. (2022). Sodium butyrate ameliorates neurotoxicity and exerts anti-inflammatory effects in high-fat diet-fed mice. Food Chem. Toxicol..

[49] Matta F.V., Xiong J., Lila M.A., Ward N.I., Felipe-Sotelo M., Esposito D. (2020). Chemical Composition and Bioactive Properties of Commercial and Non-Commercial Purple and White Açaí Berries. Foods.

[50] Golovinskaia O., Wang C.K. (2021). Review of Functional and Pharmacological Activities of Berries. Molecules.

[51] Skrovankova S., Sumczynski D., Mlcek J., Jurikova T., Sochor J. (2015). Bioactive Compounds and Antioxidant Activity in Different Types of Berries. Int. J. Mol. Sci..

[52] Angeloni C., Pirola L., Vauzour D., Maraldi T. (2012). Dietary polyphenols and their effects on cell biochemistry and pathophysiology. Oxid. Med. Cell. Longev..

[53] Calabrò R.S., De Cola M.C., Gervasi G., Portaro S., Naro A., Accorinti M., Manuli A., Marra A., De Luca R., Bramanti P. (2019). The Efficacy of Cocoa Polyphenols in the Treatment of Mild Cognitive Impairment: A Retrospective Study. Medicina.

[54] Kuszewski J.C., Howe P.R.C., Wong R.H.X. (2020). Evaluation of cognitive performance following fish-oil and curcumin supplementation in middle-aged and older adults with overweight or obesity. J. Nutr..

[55] Carrillo J.Á., Arcusa R., Xandri-Martínez R., Cerdá B., Zafrilla P., Marhuenda J. (2025). Impact of Polyphenol-Rich Nutraceuticals on Cognitive Function and Neuroprotective Biomarkers: A Randomized, Double-Blind, Placebo-Controlled Clinical Trial. Nutrients.

[56] Tejada S., Sarubbo F., Jiménez-García M., Ramis M.R., Monserrat-Mesquida M., Quetglas-Llabrés M.M., Capó X., Esteban S., Sureda A., Moranta D. (2024). Mitigating Age-Related Cognitive Decline and Oxidative Status in Rats Treated with Catechin and Polyphenon-60. Nutrients.

[57] Travica N., D’Cunha N.M., Naumovski N., Kent K., Mellor D.D., Firth J., Georgousopoulou E.N., Dean O.M., Loughman A., Jacka F. (2020). The effect of blueberry interventions on cognitive performance and mood: A systematic review of randomized controlled trials. Brain Behav. Immun..

[58] Krikorian R., Shidler M.D., Summer S.S. (2023). Early Intervention in Cognitive Aging with Strawberry Supplementation. Nutrients.

[59] Groven S., Devillez P., Scofield R.H., Champion A., Izuora K., Basu A. (2025). Dietary Strawberries Improve Serum Antioxidant Profiles in Adults with Prediabetes: A 28-Week Randomized Controlled Crossover Trial. Antioxidants.

[60] da Silva A.B.N., de Oliveira G.M., Gallo Ruelas M., Gadelha M.S.M., de Farias Santos A.C.F., Zamora F.V. (2025). Blueberries for brainpower: A systematic review and meta-analysis with Bayesian post hoc analysis of RCTS exploring cognitive function in the elderly with prior cognitive decline. Biogerontology.

[61] Krikorian R., Shidler M.D., Nash T.A., Kalt W., Vinqvist-Tymchuk M.R., Shukitt-Hale B., Joseph J.A. (2010). Blueberry supplementation improves memory in older adults. J. Agric. Food Chem..

[62] Pan P., Skaer C.W., Stirdivant S.M., Young M.R., Stoner G.D., Lechner J.F., Huang Y.W., Wang L.S. (2015). Beneficial regulation of metabolic profiles by black raspberries in human colorectal cancer patients. Cancer Prev. Res..

[63] Flanagan E., Cameron D., Sobhan R., Wong C., Pontifex M.G., Tosi N., Mena P., Del Rio D., Sami S., Narbad A. (2022). Chronic Consumption of Cranberries (*Vaccinium macrocarpon*) for 12 Weeks Improves Episodic Memory and Regional Brain Perfusion in Healthy Older Adults: A Randomised, Placebo-Controlled, Parallel-Groups Feasibility Study. Front. Nutr..

[64] Bell L., Williams C.M. (2019). A pilot dose–response study of the acute effects of haskap berry extract (*Lonicera caerulea* L.) on cognition, mood, and blood pressure in older adults. Eur. J. Nutr..

[65] Battino M., Giampieri F., Cianciosi D., Ansary J., Chen X., Zhang D., Gila E., Forbes-Hernández T.Y. (2021). The roles of strawberry and honey phytochemicals on human health: A possible clue on the molecular mechanisms involved in the prevention of oxidative stress and inflammation. Phytomedicine.

[66] Molan A.L., Liu Z., Plimmer G. (2014). Evaluation of the effect of blackcurrant products on gut microbiota and on markers of risk for colon cancer in humans. Phytother. Res..

[67] Rendeiro C., Rhodes J.S., Spencer J.P.E. (2015). The mechanisms of action of flavonoids in the brain: Direct versus indirect effects. Neurochem. Int..

[68] Tandoro Y., Chiu H.-F., Tan C.-L., Hsieh M.-H., Huang Y.-W., Yu J., Wang L.-S., Chan C.-H., Wang C.-K. (2025). Black raspberry supplementation on overweight and Helicobacter pylori infected mild dementia patients a pilot study. npj Sci. Food.

[69] Williams R.J., Spencer J.P.E. (2012). Flavonoids, cognition, and dementia: Actions, mechanisms, and potential therapeutic utility for Alzheimer disease. Free Radic. Biol. Med..

[70] Miller K., Feucht W., Schmid M. (2019). Bioactive compounds of strawberry and blueberry and their potential health effects based on human intervention studies: A brief overview. Nutrients.

[71] Bonyadi N., Dolatkhah N., Salekzamani Y., Hashemian M. (2022). Effect of berry-based supplements and foods on cognitive function: A systematic review. Sci. Rep..

[72] Jiang H., Zhang W., Li X., Xu Y., Cao J., Jiang W. (2021). The anti-obesogenic effects of dietary berry fruits: A review. Food Res. Int..

[73] Thomas J., Garg M.L., Smith D.W. (2014). Dietary resveratrol supplementation normalizes gene expression in the hippocampus of streptozotocin-induced diabetic C57Bl/6 mice. J. Nutr. Biochem..

[74] Jeon B.T., Jeong E.A., Shin H.J., Lee Y., Lee D.H., Kim H.J., Roh G.S. (2012). Resveratrol attenuates obesity-associated peripheral and central inflammation and improves memory deficit in mice fed a high-fat diet. Diabetes.

[75] Dragano N.R.V., Marques A.Y.C., Cintra D.E.C., Solon C., Morari J., Leite-Legatti A.V., Velloso L.A., Maróstica-Júnior M.R. (2013). Freeze-dried jaboticaba peel powder improves insulin sensitivity in high-fat-fed mice. Br. J. Nutr..

[76] Figueira I., Garcia G., Pimpão R.C., Terrasso A.P., Costa I., Almeida A.F., Santos C.N. (2017). Polyphenols journey through the blood–brain barrier towards neuronal protection. Sci. Rep..

[77] Youdim K.A., Dobbie M.S., Kuhnle G., Proteggente A.R., Abbott N.J., Rice-Evans C. (2003). Interaction between flavonoids and the blood-brain barrier: In vitro studies. J. Neurochem..

[78] Lin D., Xiao M., Zhao J., Li Z., Xing B., Li X., Kong M., Li L., Zhang Q., Liu Y. (2016). An Overview of Plant Phenolic Compounds and Their Importance in Human Nutrition and Management of Type 2 Diabetes. Molecules.

[79] Bayazid A.B., Lim B.O. (2022). Quercetin Is an Active Agent in Berries against Neurodegenerative Diseases Progression through Modulation of Nrf2/HO1. Nutrients.

[80] Markočević M., Ivković M., Zloh M. (2025). Understanding the Therapeutic Potential of Quercetin and Resveratrol: Computational Insights into Antidiabetic Activity. Chem. Proc..

[81] Leri M., Natalello A., Bruzzone E., Stefani M., Bucciantini M. (2019). Oleuropein aglycone and hydroxytyrosol interfere differently with toxic Aβ_1–42_ aggregation. Food Chem. Toxicol..

[82] Lee J.-S., Cha Y.-J., Lee K.-H., Yim J.-E. (2016). Onion peel extract reduces the percentage of body fat in overweight and obese subjects: A 12-week, randomized, double-blind, placebo-controlled study. Nutr. Res. Pract..

[83] Arabi S.M., Jazinaki M.S., Chambari M., Bahrami L.S., Maleki M., Sukhorukov V.N., Sahebkar A. (2023). The effects of Quercetin supplementation on cardiometabolic outcomes: An umbrella review of meta-analyses of randomized controlled trials. Phytother. Res..

[84] Niziński P., Hawrył A., Polak P., Kondracka A., Oniszczuk T., Soja J., Hawrył M., Oniszczuk A. (2025). Potential of Quercetin as a Promising Therapeutic Agent Against Type 2 Diabetes. Molecules.

[85] Cheng N., Barfoot K.L., Le Cozannet R., Fança-Berthon P., Lamport D.J., Williams C.M. (2024). Wild blueberry extract intervention in healthy older adults: A multi-study, randomized, controlled investigation of acute cognitive and cardiovascular effects. Nutrients.

[86] Krikorian R., Skelton M.R., Summer S.S., Shidler M.D., Sullivan P.G. (2022). Blueberry Supplementation in Midlife for Dementia Risk Reduction. Nutrients.

[87] Devore E.E., Kang J.H., Breteler M.M.B., Grodstein F. (2012). Dietary intakes of berries and flavonoids in relation to cognitive decline. Ann. Neurol..

[88] Doraiswamy P.M., Miller M.G., Hellegers C.A., Nwosu A., Choe J., Murdoch D.M. (2023). Blueberry Supplementation Effects on Neuronal and Pathological Biomarkers in Subjects at Risk for Alzheimer’s Disease: A Pilot Study. J. Aging Res. Lifestyle.

[89] Wood E., Hein S., Mesnage R., Fernandes F., Abhayaratne N., Xu Y., Zhang Z., Bell L., Williams C., Rodriguez-Mateos A. (2023). Wild blueberry (poly)phenols can improve vascular function and cognitive performance in healthy older individuals: A double-blind randomized controlled trial. Am. J. Clin. Nutr..

[90] Miller M.G., Hamilton D.A., Joseph J.A., Shukitt-Hale B. (2018). Dietary blueberry improves cognition among older adults in a randomized, double-blind, placebo-controlled trial. Eur. J. Nutr..

[91] Miller M.G., Thangthaeng N., Rutledge G.A., Scott T.M., Shukitt-Hale B. (2021). Dietary strawberry improves cognition in a randomised, double-blind, placebo-controlled trial in older adults. Br. J. Nutr..

[92] Nilsson A., Salo I., Plaza M., Björck I. (2017). Effects of a mixed berry beverage on cognitive functions and cardiometabolic risk markers: A randomized cross-over study in healthy older adults. PLoS ONE.

[93] Lopresti A.L., Smith S.J., Pouchieu C., Pourtau L., Gaudout D., Pallet V., Drummond P.D. (2023). Effects of a polyphenol-rich grape and blueberry extract (Memophenol™) on cognitive function in older adults with mild cognitive impairment: A randomized, double-blind, placebo-controlled study. Front. Psychol..

[94] Dodd G.F., Williams C.M., Butler L.T., Spencer J.P.E. (2019). Acute effects of flavonoid-rich blueberry on cognitive and vascular function in healthy older adults. Nutr. Health Aging.

[95] Whyte A.R., Cheng N., Butler L.T., Lamport D.J., Williams C.M. (2019). Flavonoid-rich mixed berries maintain and improve cognitive function over a 6 h period in young healthy adults. Nutrients.

[96] Curtis P.J., van der Velpen V., Berends L., Jennings A., Haag L., Minihane A.M., Chandra P., Kay C.D., Rimm E.B., Cassidy A. (2024). Chronic and postprandial effect of blueberries on cognitive function, alertness, and mood in participants with metabolic syndrome—Results from a six-month, double-blind, randomized controlled trial. Am. J. Clin. Nutr..

[97] Whyte A.R., Rahman S., Bell L., Edirisinghe I., Krikorian R., Williams C.M., Burton-Freeman B. (2021). Improved metabolic function and cognitive performance in middle-aged adults following a single dose of wild blueberry. Eur. J. Nutr..

[98] Cheatham C.L., Johnston C.A., Juarez L.D., O’Shea C., Hanson C., Tolan N.V. (2023). Six-month intervention with wild blueberries improved speed of processing in mild cognitive impairment: A double-blind, placebo-controlled, randomized clinical trial. Nutr. Neurosci..

[99] Delaney K., Tsang M., Kern M., Rayo V.U., Jason N., Hong M.Y., Liu C., Hooshmand S. (2025). Strawberries modestly improve cognition and cardiovascular health in older adults. Nutr. Metab. Cardiovasc. Dis..

[100] Kakutani S., Watanabe H., Murayama N. (2019). Green Tea Intake and Risks for Dementia, Alzheimer’s Disease, Mild Cognitive Impairment, and Cognitive Impairment: A Systematic Review. Nutrients.

[101] Zhang N., Jing P. (2023). Red cabbage anthocyanins attenuate cognitive impairment by attenuating neuroinflammation and regulating gut microbiota in aging mice. J. Agric. Food Chem..

[102] Fan K.C., Lin C.C., Chiu Y.L., Koh S.H., Liu Y.C., Chuang Y.F. (2025). Compositional and functional gut microbiota alterations in mild cognitive impairment: Links to Alzheimer’s disease pathology. Alzheimer’s Res. Ther..

[103] O’Riordan K.J., Moloney G.M., Keane L., Clarke G., Cryan J.F. (2025). The gut microbiota-immune-brain axis: Therapeutic implications. Cell Rep. Med..

[104] Khan M.S., Ikram M., Park J.S., Park T.J., Kim M.O. (2020). Gut microbiota, its role in induction of Alzheimer’s disease pathology, and possible therapeutic interventions: Special focus on anthocyanins. Cells.

[105] Milenkovic D., Krga I., Dinel A.L., Morand C., Layé S., Castanon N. (2021). Nutrigenomic modification induced by anthocyanin-rich bilberry extract in the hippocampus of ApoE^−^/^−^ mice. J. Funct. Foods.

[106] Suresh S., Begum R.F., Singh A. (2022). Anthocyanin as a therapeutic in Alzheimer’s disease: A systematic review of preclinical evidences. Ageing Res. Rev..

[107] Lee Y.M., Yoon Y., Yoon H., Park H.M., Song S., Yeum K.J. (2017). Dietary Anthocyanins against Obesity and Inflammation. Nutrients.

[108] Geda Y.E. (2012). Mild cognitive impairment in older adults. Curr. Psychiatry Rep..

[109] Engin A. (2017). The pathogenesis of obesity-associated adipose tissue inflammation. Adv. Exp. Med. Biol..

[110] Farias G., Netto B.D.M., Boritza K., Bettini S.C., Vilela R.M., Dâmaso A.R. (2020). Impact of weight loss on inflammation state and endothelial markers among individuals with extreme obesity after gastric bypass surgery: A 2-year follow-up study. Obes. Surg..

[111] García-García I., Fernández-Andújar M., Narváez M., García-Casares N. (2023). Assessing adipokines as potential biomarkers of dementia, Alzheimer’s disease, and mild cognitive impairment: A systematic review and meta-analysis. Obes. Rev..

[112] Wallace T.C., Bailey R.L., Blumberg J.B., Burton-Freeman B., Chen C.O., Crowe-White K.M., Drewnowski A., Hooshmand S., Johnson E., Lewis R. (2020). Fruits, vegetables, and health: A comprehensive narrative, umbrella review of the science and recommendations for enhanced public policy to improve intake. Crit. Rev. Food Sci. Nutr..

[113] Musich M., Curtis A.F., Ferguson B.J., Drysdale D., Thomas A.L., Greenlief C.M., Shenker J.I., Beversdorf D.Q. (2025). Preliminary Effects of American Elderberry Juice on Cognitive Functioning in Mild Cognitive Impairment Patients: A Secondary Analysis of Cognitive Composite Scores in a Randomized Clinical Trial. Antioxidants.

[114] Sweeney M.I., Kalt W., MacKinnon S.L., Ashby J., Gottschall-Pass K.T. (2002). Feeding rats diets enriched in lowbush blueberries for six weeks decreases ischemia-induced brain damage. Nutr. Neurosci..

[115] Batista A.G., Zanzer Y.C., Marostica Junior M.R., Östman E.M. (2025). Jaboticaba peel intake improved cognitive performance, inflammatory response, and appetite regulation in healthy adults: A randomized clinical crossover trial. Food Res. Int..

[116] Bouyahya A., Omari N.E., EL Hachlafi N., Jemly M.E., Hakkour M., Balahbib A., El Menyiy N., Bakrim S., Naceiri Mrabti H., Khouchlaa A. (2022). Chemical Compounds of Berry-Derived Polyphenols and Their Effects on Gut Microbiota, Inflammation, and Cancer. Molecules.

