# Supplementary material for: Berry Consumption and Its Role in the Modulation of Obesity and Mild Cognitive Impairment
Source: Nutrients. 2026 Feb 19;18(4):674. doi: 10.3390/nu18040674 (PMC12943443; doi:10.3390/nu18040674)
Supplement: Supplementary file 1 [file nutrients-18-00674-s001.zip › nutrients-3993931-supplementary.pdf]

Section	Item	Checklist item	Location in manuscript
Title	1	Identify the report as a systematic review	Title
Abstract	2	Provide a structured summary	Abstract
Introduction	3	Describe the rationale for the review	Introduction
Introduction	4	Provide an explicit statement of objectives	Introduction
Methods	5	Specify eligibility criteria	Methods
Methods	6	Specify information sources	Methods
Methods	7	Present full search strategy	Table 1 and figure 4 (Methods)
Methods	8	Describe selection process	Methods
Methods	9	Describe data collection process	Methods
Methods	10	Define data items	Methods
Methods	11	Describe risk of bias assessment	Methods
Methods	12	Describe effect measures (if applicable)	Not applicable
Methods	13	Describe synthesis methods	Methods
Methods	14	Describe reporting bias assessment	Not assessed
Methods	15	Describe certainty assessment	Not assessed
Results	16	Describe study selection	Results
Results	17	Cite excluded studies	Methods-figure 4
Results	18	Present characteristics of included studies	Results
Results	19	Present risk of bias in studies	Results
Results	20	Present results of individual studies	Results
Results	21	Present synthesis of results	Results
Discussion	22	Interpret results in context	Discussion
Discussion	23	Discuss limitations of evidence	Discussion
Discussion	24	Discuss implications	Discussion
Other	25	Provide registration information	Not registered

Section	Item	Checklist item	Location in manuscript
Other	26	Describe protocol deviations	Not applicable
Other	27	Describe availability of data	Data availability statement
